# Reduction of mRNA export unmasks different tissue sensitivities to low mRNA levels during *Caenorhabditis elegans* development

**DOI:** 10.1371/journal.pgen.1008338

**Published:** 2019-09-16

**Authors:** Angelina Zheleva, Eva Gómez-Orte, Beatriz Sáenz-Narciso, Begoña Ezcurra, Henok Kassahun, María de Toro, Antonio Miranda-Vizuete, Ralf Schnabel, Hilde Nilsen, Juan Cabello

**Affiliations:** 1 CIBIR (Center for Biomedical Research of La Rioja), Logroño, La Rioja, Spain; 2 Department of Clinical Molecular Biology, Institute of Clinical Medicine, University of Oslo and Akershus University Hospital, Lørenskog, Norway; 3 Instituto de Biomedicina de Sevilla, Hospital Universitario Virgen del Rocío/CSIC/Universidad de Sevilla, Sevilla, Spain; 4 Institute of Genetics, Technische Universität Braunschweig, Germany; University of Pennsylvania School of Medicine, UNITED STATES

## Abstract

Animal development requires the execution of specific transcriptional programs in different sets of cells to build tissues and functional organs. Transcripts are exported from the nucleus to the cytoplasm where they are translated into proteins that, ultimately, carry out the cellular functions. Here we show that in *Caenorhabditis elegans*, reduction of mRNA export strongly affects epithelial morphogenesis and germline proliferation while other tissues remain relatively unaffected. Epithelialization and gamete formation demand a large number of transcripts in the cytoplasm for the duration of these processes. In addition, our findings highlight the existence of a regulatory feedback mechanism that activates gene expression in response to low levels of cytoplasmic mRNA. We expand the genetic characterization of nuclear export factor NXF-1 to other members of the mRNA export pathway to model mRNA export and recycling of NXF-1 back to the nucleus. Our model explains how mutations in genes involved in general processes, such as mRNA export, may result in tissue-specific developmental phenotypes.

## Author summary

The Central Dogma of Biology schematically highlights the transmission of genetic information stored in DNA, through RNA, to the formation of proteins. This general flow implicates RNA export from the nucleus to the cytoplasm and proper protein localization within the eukaryotic cell. Ultimately, proteins are the cell’s structural and catalytic functional units. As a result, cells differentiate into one cell type or another (such as epithelial, muscle, neuron…) and exhibit specific shape and functionality. Here we describe, in a *C*. *elegans* model, how mutations in genes involved in a general and ubiquitous mechanism, such as mRNA export, may result in tissue-specific developmental phenotypes that show up in processes that are highly demanding of cytoplasmic transcripts like epithelialization and gamete formation. A deep understanding of the mechanisms underlying the "connectors" shown in the Central Dogma of Biology is key both to unravel the general genetic control of an organism’s development and, at the same time, contribute to a better understanding of tissue-specific diseases.

## Introduction

Cell differentiation and morphogenesis rely on the expression of specific genes that are translated into proteins in specific sets of cells to ensure the correct formation of the organs and body plan. The physical separation between genomic DNA and the cytoplasm in eukaryotic cells makes it necessary to export RNA through the nuclear envelope (NE) [1,2,3,4,5]. This nucleo-cytoplasmic transport is highly conserved [6] and our understanding of its mechanism comes from a variety of model organisms including yeast, nematodes, fruit flies and vertebrates [4,5,7,8].

mRNA biogenesis and export are tightly coordinated by sequential assembly of appropriate ribonucleoprotein complexes named the THO complex (named after the yeast tho2 subunit was identified as a suppressor of the Transcriptional defect of Hpr1 by Overexpression), the TREX (TRanscription EXport) complex and the THSC/TREX-2 (Transport/export complex 2) complex [4,5,9,10,11]. Briefly, during transcription, a group of proteins called the THO complex is recruited to chromatin. This complex is needed for transcription elongation, mRNA export and genome integrity [12,13]. The metazoan THO complex contains THOC1/2/3/5/6/7 (THO complex in yeast: Hpr1, Tho2, Mtf1 and Thp2) [14]. Next, additional proteins UAP65, Aly/REF and CIP29 (Sub2p, Yra1p and Nab2 in yeast) bind the THO subunits to build the transcription-export complex (TREX complex) which couples transcription with mRNA export [15].

**Fig 1:**
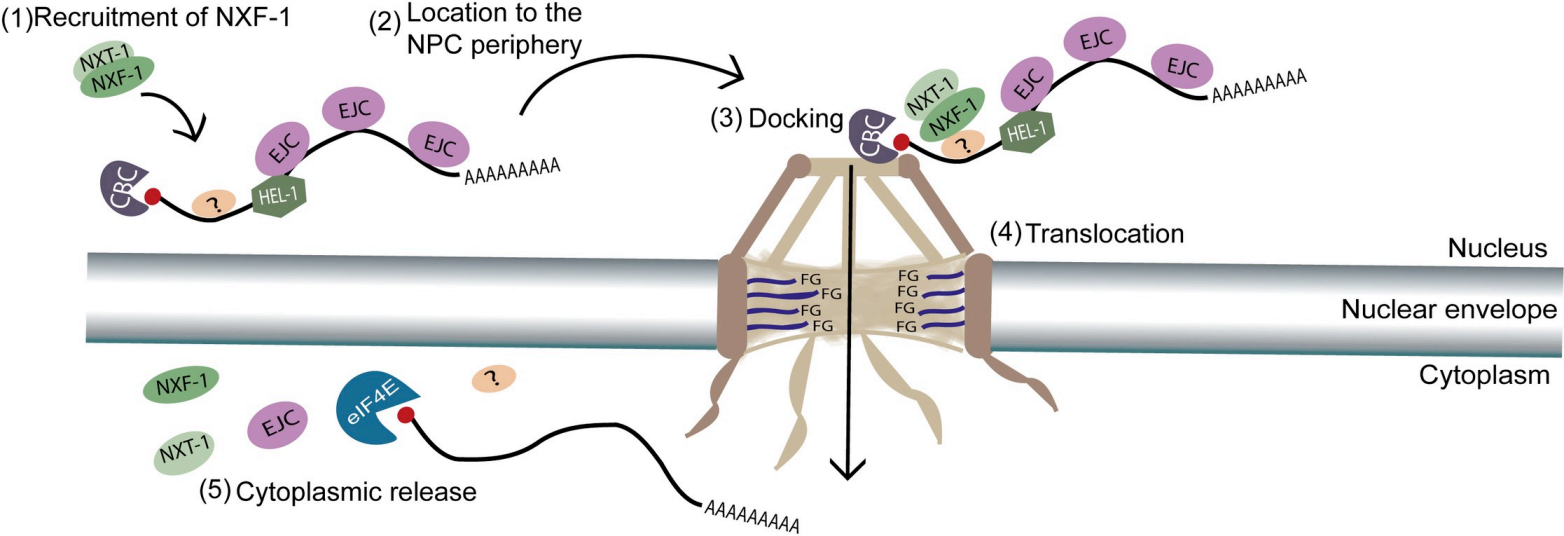
Schematic overview of nuclear mRNA export. The primary steps in mRNA export are shown. (1) Recruitment of the NXF-1–NXT-1 (NXF1-p15) heterodimer to mRNA (2) nascent mRNP-NXF-1 is docked to the nuclear pore, (3) followed by translocation (4) and cytoplasmic release (5) at the cytoplasmic filaments.

After the messenger ribonucleoprotein (mRNP) has been generated, the conserved nuclear RNA export factor 1 (NXF1/TAP) is recruited through direct interaction with several TREX components [2,16]. NXF1 family export factors are composed of multiple domains. At the N terminus is the RNA recognition motif (RRM) [17]. Next, a leucine-rich repeat domain (LRR) is required for NXF1-mediated export [18]. This domain is followed by a nuclear transport factor 2, NTF2-like domain, that heterodimerizes with a protein known as p15 or NXT [19,20,21,22]. Efficient mRNA export from the nucleus to the cytoplasm requires the formation of this complex. The remaining C-terminal domain, TAP, also known as the NXF1 ubiquitin-associated domain (UBA), permits translocation through the central channel of the nuclear pore complex (NPC) by interacting with FG-Nups (phenylalanine-glycine (FG) reach nucleoporins) [6,20,21,23,24]. Finally, the THSC/TREX-2 (transport/export complex 2), binds the mRNP to the nucleoplasmic side of the NPC. The transit of mRNP through the nuclear pore is mediated by direct interaction of NXF1-p15 with the nucleoporins that line the pore [25]. Once in the cytoplasm, mRNA can be stored in large ribonucleotide protein particles (RNP), as happens in the so-called germ granules (known as P granules in *C*. *elegans*) or it can be directly translated into proteins [26,27,28] (Fig 1).

Over the last decades, *C*. *elegans* has emerged as a powerful model for studying cell differentiation and morphogenesis. *C*. *elegans* has a simple body plan. Schematically, it can be divided into two cylindrical layers of tissues and organs separated by a fluid-filled space (pseudocoelom). From outside to inside, the outer layer constitutes the body wall, which consists of the cuticle and an epithelium called the epidermis (also known as the hypodermis) [29], the excretory system, neurons and muscles. The inner system is comprised of the gonad and another epithelial tube composed of the pharynx and intestine [30]. The anterior portion of the pharynx and the external epidermis remain linked by nine cells called the arcade cells. Absence of this arcade cell epithelium leads to a Pun (pharynx unattached) phenotype where the pharynx detaches from the mouth during development and forms a confined cell cluster in the interior of the animal [30,31,32,33].

Several pathways contribute to cell fate specification and epithelialization of arcade cells. It occurs after the epidermis and pharynx have epithelialized. The process is very fast (less than 10 minutes), during mid-embryogenesis after the embryonic cell divisions are complete [33]. Recent studies show that PAR-6/PARD6A is required for polarizing the arcade cells to define typical apical and basolateral domains [34,35,36]. In *C*. *elegans* epithelial cells, both domains are separated by the adherens junctions (CeAJ). Thus, the CeAJ contains proteins that mediate adhesion such as HMR-1/cadherin, HMP-1/-catenin, HMP-2/-catenin, and VAB-9/claudin [37,38]. In addition, DLG-1/Discs large and the coiled-coil protein AJM-1, are also part of the CeAJ although they are located slightly more basally [39,40].

In this study we show that reducing mRNA export strongly affects epithelial formation and germline proliferation in *C*. *elegans*. Previous studies revealed that both processes require specific gene expression programs. Our findings indicate the existence of feedback mechanisms that activate expression of specific genes to compensate for the lack of mRNAs in the cytoplasm such as those involved in mRNA export or cytoskeletal rearrangements. Finally, we suggest a model to explain the mRNA export pathway and recycling of the export factor NXF-1 back to the nucleus in *C*. *elegans* to close the export cycle.

## Results

### Identification of a *C*. *elegans nxf-1* mutant with pharyngeal and morphogenetic embryonic defects

**Fig 2:**
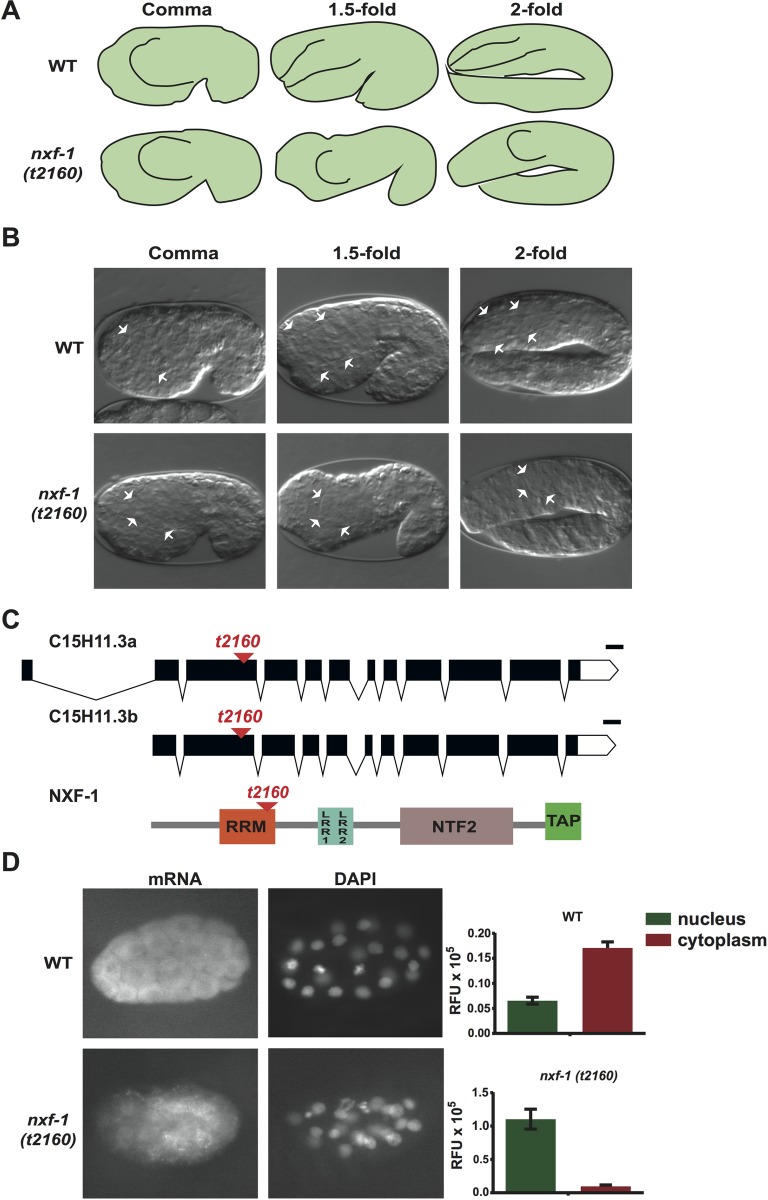
*nxf-1(t2160*ts*)* mutant shows unattached Pun pharynx. **(A)** Schematic overview of pharyngeal morphogenesis during WT and *nxf-1(t2160*ts*)* embryogenesis. **(B)** Differential interference contrast (DIC) image of comma, 1.5-fold and 2-fold embryos showing WT embryo elongation with pharynx attached to the buccal cavity and similar images of *nxf-1(t2160*ts*)* mutant embryos in which the pharynx failed to reach the buccal cavity (unattached phenotype, Pun). White arrows point to the basement membrane surrounding the developing pharynx. **(C)** Genomic organization of the *nxf-1* gene and NXF-1 protein domains. Red arrowhead indicates the *t2160*ts mutation. **(D)** mRNA visualized by FISH with a poly-dT oligonucleotide conjugated with Cy3. Strong accumulation of poly(A)mRNA in the *nxf-1(t2160*ts*)* nucleus contrasts with the WT nucleus where no signal accumulation was distinguishable, and mRNA concentrates in the cytoplasm. mRNA accumulation is measured as relative fluorescent units (RFU) in the cellular nucleus and cytoplasm of FISH-stained embryos.

To discover genes involved in embryonic morphogenesis, we performed a genetic screen for embryonic lethal worms with the pharynxes unattached to the mouth (Pun phenotype). Thus, we identified a thermo-sensitive (ts) mutant allele, *t2160*, whose embryos arrested at late stages with a highly penetrant Pun phenotype (87.5% (n = 176)) and body elongation defects (81% (n = 60)) (Fig 2A and 2B, Table 1). Three experimental lines demonstrated that *t2160*ts is an allele of the *C*. *elegans* nuclear export factor 1 (*nxf-1*) [41]. First, using the whole genome sequencing approach (WGS) and CloudMap/Hawaiian Variant Mapping (http://usegalaxy.org/cloudmap)[42], we identified a homozygous A-to-G transition at the 14414501 position of chromosome V, in the C15H11.3/*nxf-1* gene that caused a VAL to ALA substitution. The *t2160*ts mutation was located in the RRM (RNA recognition motif) domain of NXF-1 (Fig 2C). Second, *t2160*ts failed to complement a knockout deletion of the *nxf-1*(*ok1281*) gene. Third, the *t2160*ts embryonic lethality was successfully rescued with a plasmid containing 1096 bp of the gene promoter, the *nxf-1* genomic region and 1313 bp of the 3' UTR (S1 Fig). To determine whether the ts effect of the VAL to ALA substitution was likely due to synthesis or to folding [43] we performed a ts curve assay during embryo development (S2 Fig). In an up-shift curve, worms grown to adulthood at 15°C (permissive temperature) were allowed to lay eggs which were sequentially shifted to 25°C (restrictive temperature). In those embryos, the maternal product was therefore synthesized at the permissive temperature. However, 100% of those embryos died when shifted to 25°C at the two-cell stage, and the lethality did not fall under 50% until mid-embryogenesis when most cell divisions and epithelialization are completed. The maternal product is enough to complete the development of a maternally rescued homozygous embryo at 25°C from a heterozygous *nxf-1*(*t2160*ts)(+/-) hermaphrodite mother. Together with the down-shift curve, these results show the NXF-1 requirement during embryonic cell proliferation, differentiation and morphogenesis and strongly indicate that *t2160*ts mutation alters the protein conformation when exposed to 25°C.

**Table 1:** Phenotypes observed in *nxf-1* mutants and after RNAi of mRNA export factors and EJC core proteins.

Mutant/RNAi*	Phenotype	Pun %	Epidermal defects %
*nxf-1(ok1281)*	zygotic larval lethal	-	-
*nxf-1(t2160)*	maternal late embryonic lethality	87.5	81
*nxf-1(ok1281/t2160)*	Sterile	-	-
RNAi *nxf-1* L1	arrested L2	-	-
RNAi *nxf-1* L4	early embryonic lethality	-	-
"milder" RNAi *nxf-1* L4	late embryonic lethality	71	87
RNAi *nxt-1* L1	sterile; protruding vulva	-	-
RNAi *nxt-1* L4	early embryonic lethality	-	-
"milder" RNAi *nxt-1* L4	late embryonic lethality	62	65.6
RNAi *hel-1* L1	arrested L2	-	-
RNAi *hel-1* L4	late embryonic lethality	27	100
RNAi *mag-1* L1	late embryonic lethality	-	98
RNAi *rnp-4* L1	late embryonic lethality	-	98

* *nxf-1(t2160) t*emperature-sensitive allele was grown at the permissive temperature (15°C) until the L4 larval stage and then moved to the restrictive temperature of 25°C overnight. The next day, adult worms were dissected and young embryos were left to develop at 25°*C* overnight, then scored under the microscope. For *nxf-1(ok1281/t2160)*, F1 L1 larval stage worms were separated onto new plates and grown at 25°C. Bacterial RNAi clones of *nxf-1* and *nxt-1* were diluted with L4440 at a 1:1 concentration for a “milder” *RN*Ai effect. Worms were grown at 15°C and the next day, embryos showing morphogenetic phenotypes were scored. Embryo phenotypes were assessed under the microscope using DIC optics.

Since the *t2160*ts mutation affects the *nxf-1* gene, we decided to check intracellular mRNA distribution. To examine the polyadenylated RNA localization, we performed FISH (fluorescent in situ hybridization) analysis. Hybridization with an oligo-dT probe against poly(A) showed mRNA preferentially accumulated in the nucleus rather than the cytoplasm of *nxf-1(t2160*ts*)* mutants. In contrast, the signal was mostly dispersed in the cytoplasm of wild-type (WT) embryo cells (Fig 2D). This indicates that *t2160*ts is a loss-of-function mutation in the *nxf-1* gene that impairs mRNA export. A phenotypic analysis of the available alleles and *nxf-1* RNAi reveals that the *t2160*ts mutation leads to reduced activity but is not a null allele of *nxf-1*. *nxf-1 (ok1281)* knockout maternally rescued or *nxf-1* RNAi-fed L1 larvae led to larval arrest, whereas *nxf-1 (t2160*ts*)* or RNAi performed under mild conditions (RNAi diluted with L4440 bacterial RNAi empty vector at a 1:1 ratio) led both to the same embryonic Pun phenotype and body elongation defects in the F1 embryos. Heterozygous worms *ok1281/ t2160*ts exhibited an intermediate phenotype: they reached adulthood but were sterile (Table 1).

**Fig 3:**
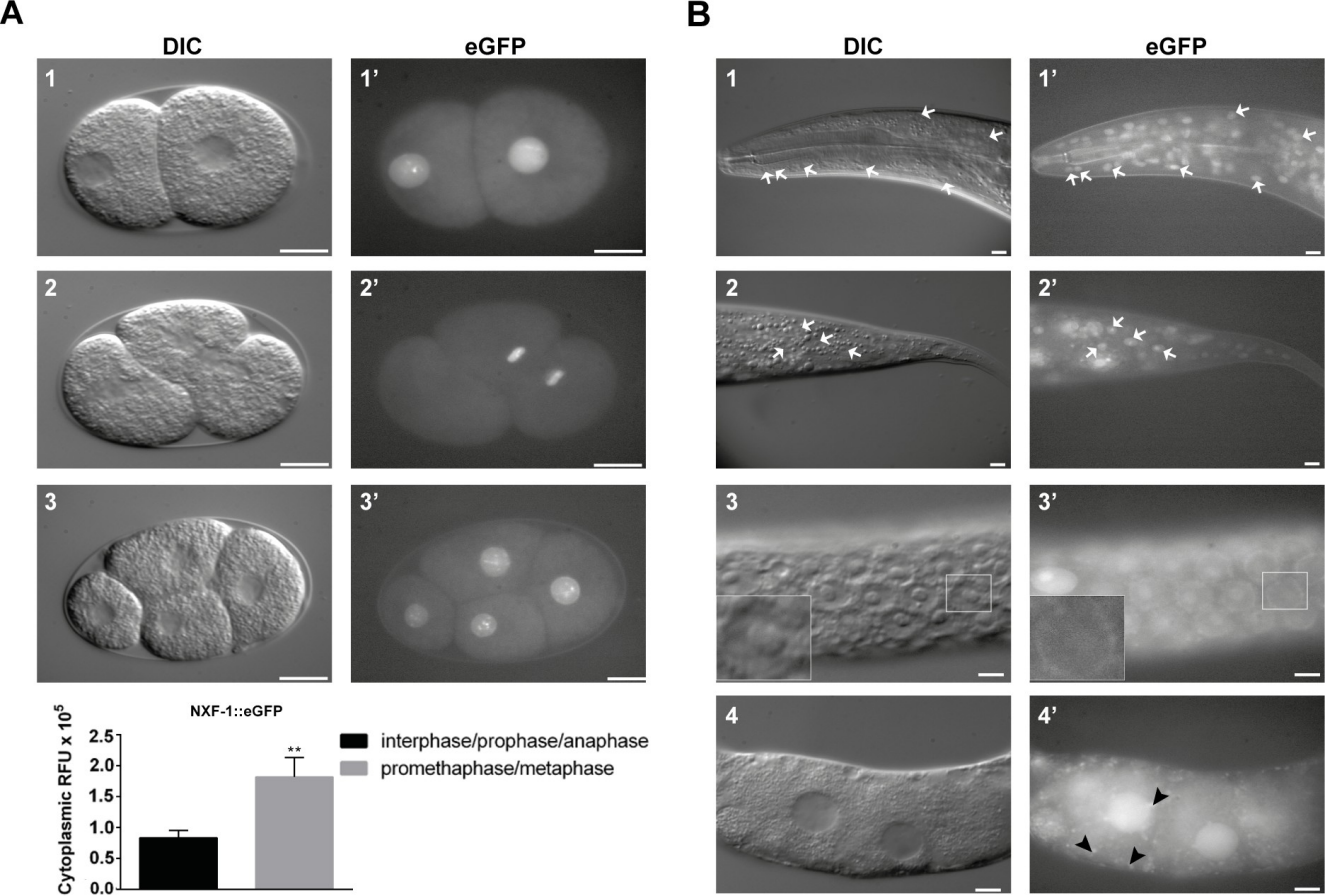
NXF-1 is concentrated in the nucleus. **(A)** (top panel) NXF-1::GFP expression in the early two-cell stage embryos (A1, A1') during cell division of the AB blastomere (A2, A2'), and in the four-cell stage (A3, A3'). Bottom panel shows quantification of the NXF-1 diffused into the cytoplasm during cell division. **(B)** NXF-1 expression in *C*. *elegans* adult somatic cells. Pictures show details of the head (B1, B1'), tail (B2, B2'), gonad (B3, B3') and oocytes (B4, B4'). White arrows indicate cellular nuclei. Black arrowheads indicate cytoplasmic granules. A detail of the localization of NXF-1 in the nuclear envelope of gonadal nuclei is shown in B3, B3' inset. Scale bar: 10μm.

To check NXF-1 localization *in vivo* and throughout development, we created transgenic lines expressing NXF-1 fused to 3xFLAG and eGFP (enhanced green fluorescent protein) (S1B Fig). The transgene successfully rescued the *nxf-1*(*t2160*ts) mutation indicating that the construct was functional and did not change the function of NXF-1. eGFP expression was detected in all stages of the *C*. *elegans* life cycle. As expected for a nuclear export factor, NXF-1::3xFLAG::eGFP showed nuclear localization during embryogenesis, larval and adult stages. NXF-1 showed dynamic localization during cell division: it was detected as nuclear but diffused to the cytoplasm during mitosis (S1 Movie). In addition, NXF-1::3xFLAG::eGFP was detected in granules in the oocyte cytoplasm (Fig 3).

**Fig 4:**
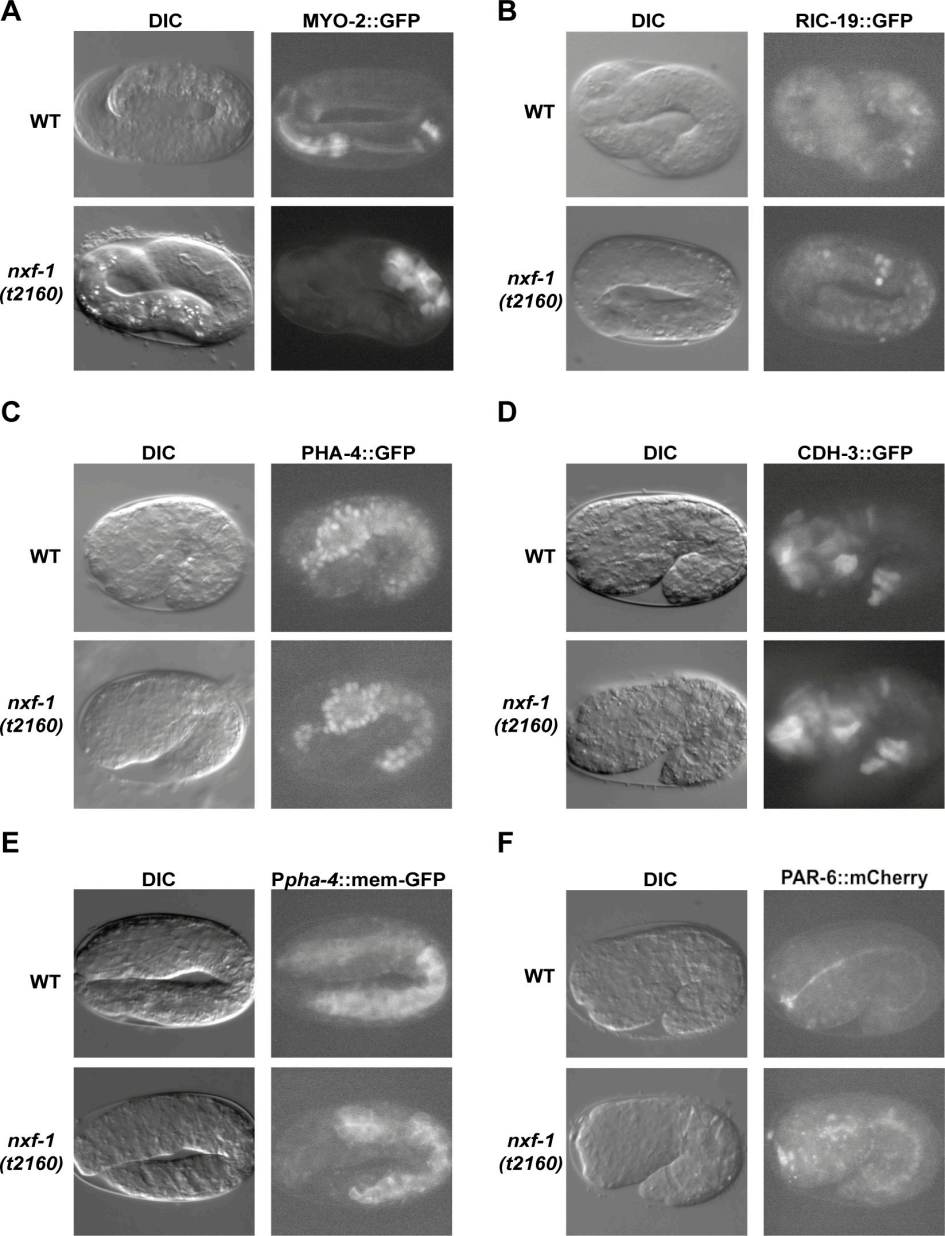
*nxf-1*(*t2160*ts) Pun pharynxes show normal tissue differentiation but failed arcade polarization. **(A)** Representative three-fold WT and *nxf-1(t2160*ts*)* embryos expressing pMYO-2::GFP reporter in pharyngeal muscle and **(B)** RIC-19::GFP expression in M1 and M2 neurons in three-fold embryos. **(C)** WT and *nxf-1(t2160*ts*)* embryos expressing PHA-4::GFP and **(D)** CDH-3::GFP reporter in the arcade cells, lateral epidermal cells and the seam cells. **(E)** Representative images of P*pha-4*::membrane–GFP reporter expression and **(F)** PAR-6::GFP shows a clear cell polarization in WT but a mislocalized expression in *nxf-1(t2160*ts*)* embryonic epithelia. Corresponding DIC (left) and fluorescence images (right) are paired for each embryo. Anterior is left, dorsal is up.

To assess the developmental defects of *nxf-1*(*t2160*ts) embryos, we performed 4D microscopy and compared it to that of WT N2 embryos (S2 Movie). Since the *nxf-1*(*t2160*ts) mutant is temperature sensitive, worms (WT and mutant) were grown at 15°C. Then, the worms where transferred to 25°C degrees and allowed to grow overnight (O/N) prior to selecting the embryos. The Pun pharynxes of the *nxf-1*(*t2160*ts) mutant embryos displayed several characteristics that are consistent with normal tissue differentiation, such as the presence of a distinct pharyngeal lumen and sustained rhythmic pumping. In further support of these results, we observed strong expression of several GFP markers indicating the presence of differentiated muscle (P*myo-2*::GFP) [44], and neurons (P*ric-19*::GFP) [45] in the Pun pharynxes (Fig 4A and 4B).

In addition to these transgenes, we tested the expression of the *pha-4* gene. The *pha-4* transcription factor is the central selector regulator gene for the *C*. *elegans* pharynx and its activity is essential for all pharyngeal development [46]. PHA-4 determines the identity and morphogenetic program of all the pharyngeal precursors by directly regulating many genes expressed in the pharynx and arcade cells at different time intervals [36,47,48,49,50,51,52,53,54]. *pha-4* expression was detected in the nuclei of the intestine, pharynx and arcade cells, both in the *nxf-1*(t2160*ts*) mutant and WT. All nine arcade nuclei could be identified and were located approximately between the pharyngeal and epidermal cells (Fig 4C), indicating that developmental programs properly differentiate pharyngeal cells in the *nxf-1*(t2160*ts*) mutant. Furthermore, normally expressed CDH-3::GFP [55] in the developing arcade cells, lateral epidermal cells and seam cells suggested proper differentiation of epithelial cells (Fig 4D).

In contrast, expression of the *pxIs10* [*P*pha*-4*::GFP::CAAX + (pRF4) *rol-6(su1006*)] transgene that generates a GFP fused to the isoprenylation sequence (CAAX) of *mig-2* [56], under control of the *pha-4* promoter to drive GFP to the plasma membrane of the same cells [30,53], revealed that although the intestinal and pharyngeal cell membranes glowed in both WT and mutant embryos, fluorescence was not detected in the arcade cell membrane of the mutant (Fig 4E). This indicated the existence of membrane or cortex defects specifically in the arcade cells of the *nxf-1* (t2160*ts*) mutant.

To further assess whether epithelialization defects in arcade cells caused the Pun phenotype in *nxf-1*(t2160*ts*) mutants, we monitored the localization of the fluorescent reporter for the *C*. *elegans* apical surface polarization protein PAR-6/Par-6 and the expression and localization of *C*. *elegans* apical junction (CeAJ) components (Fig 4F, S3A Fig): the classical cadherin-catenin complex (HMR-1/E-cadherin, JAC-1/p120-catenin, HMP-1/alpha-catenin) and the more basal AJM-1, DLG-1/disk large complex and SAX-7/L1CAM that has been proposed to function as a transmembrane component of this complex (S3 and S4 Figs) [57]. Partial loss of NXF-1 activity affected normal expression of CeAJ proteins. We detected a strong increase in DLG-1::dsRed expression and a moderate decrease of AJM-1::GFP, SAX-7::GFP and HMR-1::GFP expression in both the epidermis and the gut, that may be a direct consequence of the less efficient export of transgenic mRNA in the *nxf-1* (t2160*ts*) mutant. However, the more dramatic change in *nxf-1*(t2160*ts*) mutants was the absence of apical junctions in the arcade cells and the mislocalization of the PAR-6 polarity protein that shows an ectopic and non-polarized localization in arcade cells and also, to a lesser degree, in pharyngeal and intestinal cells as occurs in WT (Fig 4F, S5 Fig), indicating that NXF-1 is essential for arcade cells to form a polarized epithelium. In addition, proper epidermal morphogenesis was disrupted, and some cells failed to adopt their normal elongated form (Fig 4, S3 Fig, S4 Fig).

Finally, to determine whether other epithelia, such as intestine, could also be affected to a lesser extent, we analyzed WT and *nxf-1* (t2160*ts*) L1 larvae intestinal morphology. Although the mutants showed a fully developed intestine, their morphology was not completely normal and showed a wider lumen than in the WT, all along the gut duct. This defect was already detectable during embryogenesis (S6 Fig), indicating that although less sensitive than arcade and hypodermal cells, intestinal epithelia is also affected by limited mRNA export.

In summary, a partial loss of activity in the *nxf-1*(t2160*ts*) mutant predominantly affects epithelial tissues (mainly arcade cells) causing pharynx attachment defects and body elongation arrest by affecting cell-cell membrane contacts but not cell differentiation.

### mRNA export machinery is essential for pharyngeal and epidermal morphogenesis

**Fig 5:**
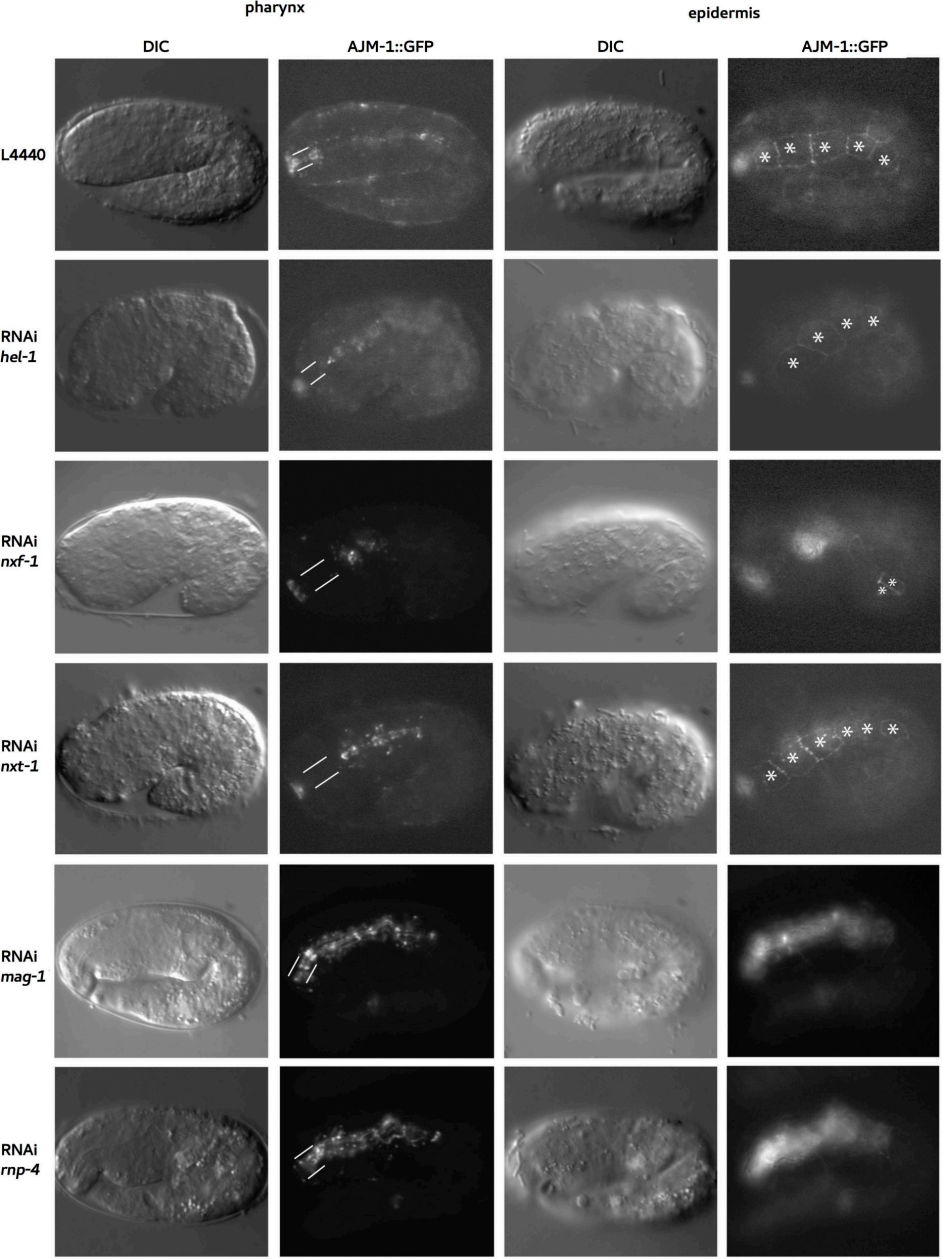
mRNA export components NXF-1/NXF1, NXT-1/NXT1 and HEL-1/UAP56 are required for *C*. *elegans* foregut tubulogenesis and epidermal morphogenesis where EJC core components RNP-4/Y14 and MAG-1/Mago-nashi are essential for *C*. *elegans* epidermal morphogenesis but dispensable for pharyngeal morphogenesis. Worms expressing AJM::GFP reporter in the L4 stage were fed with RNAi clones of *hel-1*, *nxf-1*, *nxt-1*, *mag-1* and *rnp-4*. RNAi clones of *nxf-1* and *nxt-1* were diluted with L4440 at a 1:1 concentration for a “milder” RNAi effect. Worms were grown at 15°C and images were taken the next day. Embryos depleted of *nxf-1*, *nxt-1* and *hel-1* show pharyngeal and hypodermal defects (asterisks). WT, *rnp-1(RNAi)* and *mag-1(RNAi*) arcade cells (between white lines) expressed AJM::GFP, illustrating that they are epithelialized.

To discern whether the unattached pharynx and body elongation defects observed in *nxf-1*(*t2160*ts) mutants were due to a specific function in morphogenesis or to impaired mRNA export in tissues with a high demand of mRNA, we knocked down other mRNA export machinery components and evaluated both phenotypes. Thus, we depleted NXT-1/p15 and the DEAD-box helicase, HEL-1/UAP56 by RNAi. NXT-1/p15 is an ortholog of the Ran-GDP-binding nuclear transport factors NXT1 and NXT2, that heterodimerize with NXF-1 to bind to nucleoporins and facilitate export of poly(A) RNA [2,58,59]. HEL-1/UAP56 is a DEAD-box helicase, essential for mRNA export in *C*. *elegans* [59,60] (Fig 5).

L1 larvae of the strain ST65 (ncIs13[*ajm-1*::GFP]) [61], expressing AJM-1::GFP, fed with RNAi clones of the *nxf-1* and *hel-1* genes, arrested at the L2 stage. L1 animals depleted of NXT-1 reached adulthood but were sterile and 50% of them showed protruding vulva (S7 Fig). When the RNAi experiment started at the L4 stage, all the worms progressed to adulthood and laid eggs that arrested at early embryonic stages [41,59]. To obtain a partial reduction of the mRNA export activity, we performed RNAi experiments by feeding L4 stage worms with bacterial *nxf-1* and *nxt-1* RNAi diluted with L4440 (bacterial RNAi empty vector) at a 1:1 ratio for a “milder” RNAi effect. F1 embryos developed further and died at later stages. 71% (n = 28) of the *nxf-1* RNAi embryos, 62% (n = 29) of the *nxt-1* RNAi embryos and 27% of the *hel-1* RNAi (L4 normal conditions) embryos showed unattached pharynxes with missing expression of AJM-1::GFP in arcade cells and body elongation defects (Fig 5, Table 1).

Thus, we concluded that inhibition of the mRNA export machinery by RNAi depletion of its individual components leads to a catastrophic arrest during development. In contrast, a partial reduction in mRNA export predominantly affects epithelial tissues, mainly arcade cells, causing pharynx attachment defects and body elongation arrest.

To assess whether other proteins involved in mRNA biogenesis and processing also affect embryonic morphogenesis in *C*. *elegans*, we knocked down components of the exon junction complex (EJC) and scored the unattached pharynx and body elongation defects. The EJC complex remains stably bound within mRNPs and serves as a binding platform for factors involved in mRNA packaging, export, translation and nonsense-mediated decay (NMD). Depletion of *C*. *elegans* EJC has a partial effect on mRNA splicing fidelity [62]. This complex provides a link between several steps of the mRNA life cycle [reviewed in: 63,64,65].

L1 larvae of the transgenic strain ST65 (ncIs13[*ajm-1*::GFP]) [61], expressing AJM-1::GFP, were fed with RNAi clones of the genes *mag-1*/Mago-nashi and its binding partner *rnp-4*/Y14 (components of the *C*. *elegans* EJC) [66]. Depletion of these two genes caused lethality of the F1 embryos which arrested with elongation defects [this study,59,66]. Depletion of RNP-4/Y14 did not cause nuclear accumulation of poly(A) RNAs, suggesting that *C*. *elegans* Y14 orthologue plays an essential role in *C*. *elegans* development, but is not directly associated with mRNA export [59]. The AJM::GFP reporter showed that the hypodermis was disorganized in these embryos. In contrast, pharyngeal and intestinal tissues were evident in those arrested embryos. The CeAJ in the foregut was properly formed and the pharynx was completely elongated (Fig 5).

Our results indicate that whereas the epidermis is highly sensitive to different processes affecting mRNA metabolism, such as biogenesis, processing or export, arcade cells are specifically more sensitive to mRNA export defects. This suggests the existence of different mechanisms for epithelialization or different levels of mRNA requirements for the different types of epithelia during morphogenesis.

### Mutation of *nxf-1* affects cell proliferation and DNA damage response in the *C*. *elegans* germline

**Fig 6:**
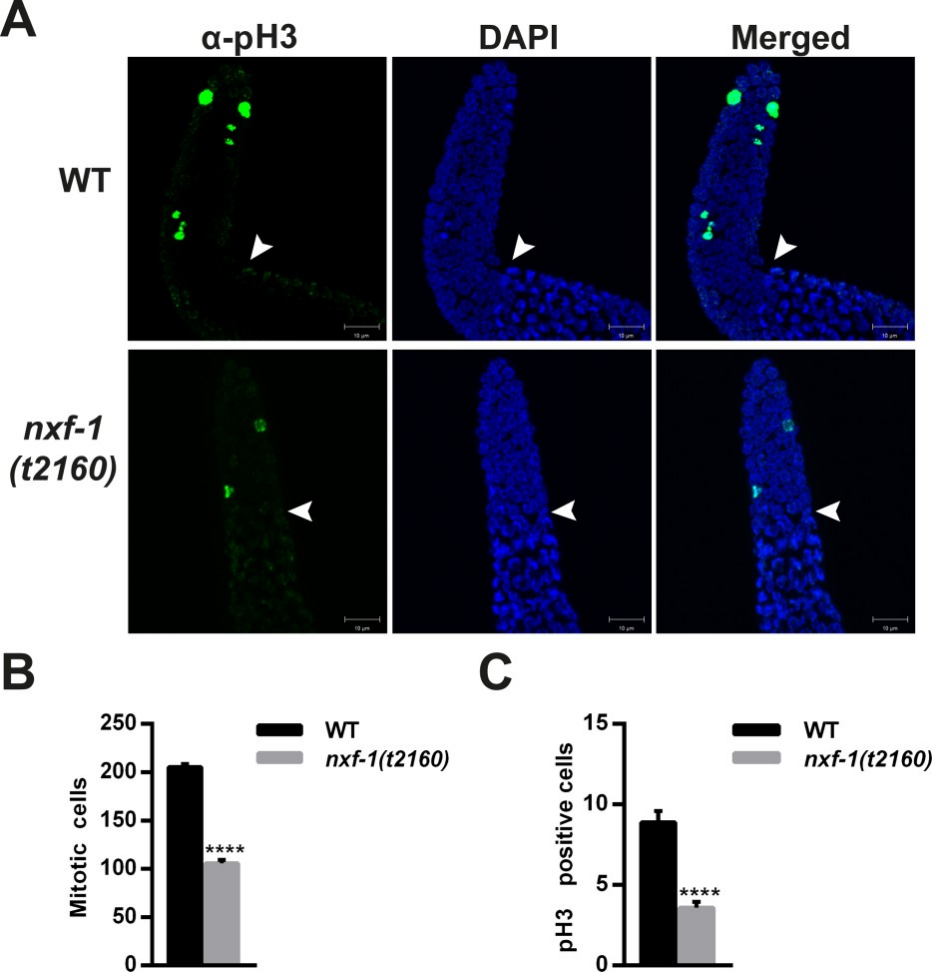
*nxf-1(t2160*ts*)* gonads show a reduced mitotic region with fewer cells in the M phase. **(A)** Representative pictures of the α-pH3 immunostained gonads. N2 (WT) and *nxf-1(t2160*ts*)* worms were synchronized. Gonads were dissected, fixed, immunostained with α-pH3 and counterstained with DAPI. **(B)** Mitotic cell quantification in the gonadal mitotic region of WT and *nxf-1(t2160*ts*)* background. **(C)** pH3 positive cell quantification in the gonadal mitotic region of WT and *nxf-1(t2160*ts*)* background. The number of pH3 positive cells and mitotic cells was manually counted in Z-Stack. Student’s t-test; ***P<0.0001. n = 20 gonads. Scale bar: 10μm.

To further explore the consequences of reducing the activity of *nxf-1* in other tissues, we expanded the analysis to the *C*. *elegans* germline. Oocyte production requires high levels of transcription and translation to accumulate enough maternal product for embryonic development [67,68]. DAPI staining of gonads at the one-day adult stage shows that the number of mitotic germ cells was strongly reduced in the *nxf-1*(*t2160*ts) mutant (Fig 6). N2 and *nxf-1*(*t2160*ts) worms were grown at 15°C until the L4 larval stage and then moved overnight to 25°C before scoring the gonad nuclei. The mitotic region of *nxf-1*(*t2160*ts) gonads had 105.9±3.4 nuclei (mean±standard error/SE) (n = 20), which is half the number of nuclei in the mitotic region of WT worms 205.36±2.9 (mean±SE) (n = 11) grown under the same conditions. In *C*. *elegans*, germline proliferation is governed by GLP-1/Notch-receptor and other effectors that mediate the transition from mitosis to meiosis [69,70]. Although, we did not find significant differences between gene expression of those factors in *nxf-1*(*t2160*ts) vs WT nematodes (S8 Fig), the inefficient transport of their mRNAs to the cytoplasm could affect the extension of the mitotic region. The number of nuclei in mitosis was determined by counting phosphorylated histone H3 (pH3)-positive nuclei in dissected gonads. Immunostaining with an anti-pH3 antibody marks cells in the late M phase [71]. This reduction in phosphorylated histone H3 is not caused by a lower level of histone expression (S9 Fig) but likely reflects the less proliferative state of the *nxf-1* (*t2160*ts) mutant gonad. The reduction of the average number of mitotic cells observed in *nxf-1*(*t2160*ts) (3.58±0.36 (mean±SE) (n = 29)) versus the WT (8.88±0.69 (mean±SE) (n = 18)) further confirmed the diminished germline proliferation in the *nxf-1*(*t2160*ts) mutant (Fig 6).

**Fig 7:**
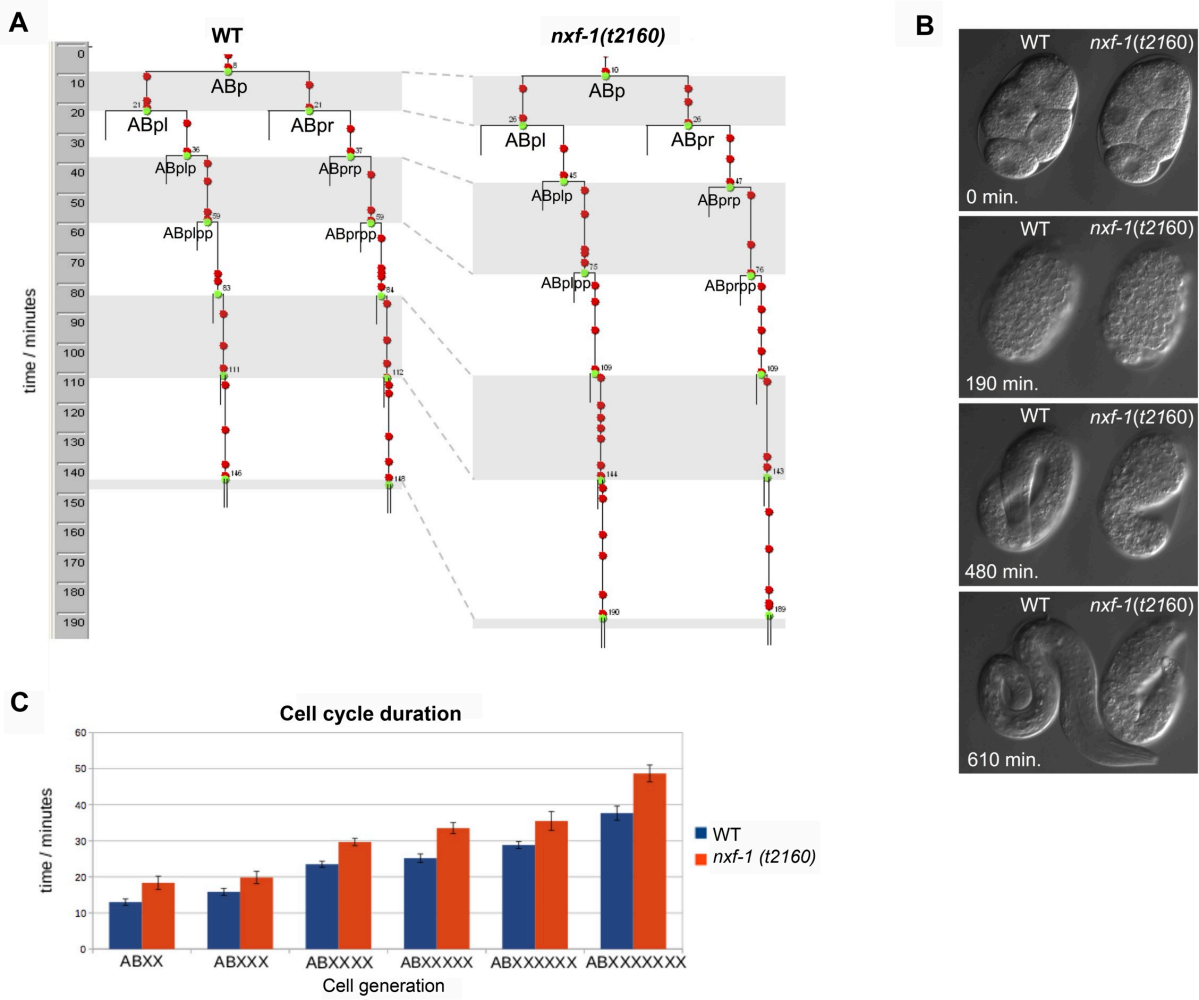
Analysis of the embryonic cell cycle progression in *nxf-1*(*t2160*ts) mutant compared to a WT embryo under the same conditions. **(A)** Representative examples of cell lineages within the AB blastomere descendants from a WT embryo and the same cells from an *nxf-1*(*t2160*ts) mutant embryo. **(B)** Cell division takes significantly longer in the mutant than in the WT. As a consequence, *nxf-1*(*t2160*ts) develops slower than a WT embryo recorded under the same conditions. **(C)** Duration of each cellular generation for WT and *nxf-1* (*t2160*ts) mutant embryos (n = 12 cells from 3 different WT and *nxf-1* (*t2160*ts) embryos).

As a canonical cell cycle progression mechanism, CDC25 dephosphorylates CDK1 to allow entry into mitosis. In *C*. *elegans*, CDK-1 is phosphorylated at the Tyr15 inhibitory residue upon DNA damage [72,73]. Phosphorylation of tyrosine (Tyr15) and threonine (Thr14) in the ATP-binding loop of CDK-1 prevents activation of the CDK/cyclin complex hindering entry into mitosis. To understand how loss of function of *nxf-1* disrupts the mitotic cell cycle, we performed immunostaining of adult gonads with antibodies against phosphorylated Tyr15 CDK-1. Our results showed an increase in Tyr15 phosphorylation of CDK-1 in the nuclei of the gonadal proliferative region of *nxf-1*(*t2160*ts) mutant animals (S10 Fig). This increase was higher than that caused by irradiation with ionizing radiation (IR) in WT animals. The absence of significant changes in the expression of *cdc-25* or *cdk-1* (S9 Fig) in *nxf-1* (*t2160*ts) vs WT and immunostaining with antibodies specific to phosphorylated proteins suggests that the reduction in germline proliferation is achieved by control of the cell cycle machinery by phosphorylation. To further check whether cell cycle impairment was an *nxf-1*(*t2160*ts)-specific phenotype or a consequence of reduced RNA export, we assayed other genes involved in RNA export. RNAi depletion of the DEAD-box helicase HEL-1/UAP56 also increased Tyr15 phosphorylation of CDK-1 in the gonadal proliferative region (S10 Fig). We next extended the analysis of cell proliferation to the cell cycle progression in the developing embryo. Consistent with the results observed in the gonad, 4D microscopic analysis shows that embryonic cell division is significantly slower in *nxf-1* (*t2160*ts) mutants compared to WT embryos under the same conditions (Fig 7). Taken together, these results suggest that RNA export reduction impairs mitotic cell cycle progression in *C*. *elegans*.

Since the germline mitotic rate was reduced upon RNA export impairment, we further investigated its role in maintenance of meiosis. The RAD-51 protein is involved in DNA repair by homologous recombination and it is a marker of double-strand breaks (DSBs) undergoing processing [74,75]. Although the expression of *rad-51* was not affected by *nxf-1*(*t2160*ts) mutation (S9 Fig), we observed a massive accumulation of RAD-51 in the pachytene/diplotene region of *nxf-1*(*t2160*ts) mutant gonads (S11 Fig). However, depletion of *hel-1* by RNAi did not produce similar RAD-51 foci (S11 Fig). This result suggests that this phenotype is not directly caused by the reduction of RNA export, but instead may reveal an additional function of *nxf-1* in genome stability. Once nuclei enter the meiotic pathway and complete the premeiotic S-phase, physiological double-strand breaks (DSBs) are generated through the action of a specialized topoisomerase enzyme SPO-11 [76]. Chromosomes align and synapse, and recombination is largely completed by late pachytene. This mechanism for initiation of meiotic recombination is conserved throughout eukaryotes. As a consequence, RAD-51 foci fail to form in *spo-11* mutants, indicative of an absence of DSBs [76]. To assay whether the increased levels of RAD-51 in *nxf-1(t2160*ts*)* mutants were due to a deregulation of SPO-11 activity, we knocked down *spo-11* by RNAi in an *nxf-1*(*t2160*ts) mutant background. Depletion of *spo-11* did not suppress the formation of RAD-51 foci in the *nxf-1*(*t2160*ts) mutant, indicating that they are independent of SPO- 11 activity (S11 Fig).

### A feed-back mechanism alters the gene expression pattern in response to low mRNA export

To gain insight into the transcriptional consequences of reducing mRNA export, we performed RNA-seq analysis of *nxf-1*(*t2160*ts) mutant worms and compared the gene expression profile to that of N2 WT worms. Since *nxf-1(t2160*ts*)* is a temperature sensitive mutant, synchronized one-day old adult-stage WT and *nxf-1(t2160*ts*)* worms grown at 15°C were shifted to 25°C for 12–16 hours before RNA extraction. Three biological replicas of each analysis were performed. RNA extraction, deep sequencing and quantitative differential expression analysis were performed as described in Material and Methods. Raw sequence data generated in this study are available at the Gene Expression Omnibus (GEO) data repository (Accession number GSE116737). Statistical analysis with the DeSeq and Edger bioinformatics algorithms showed 1117 statistically significant downregulated genes and 834 statistically significant upregulated genes in *nxf-1(t2160*ts*)* mutants vs WT (S12 Fig).

Our KEGG pathway analysis [77] of these sets of genes revealed that mRNA export reduction in the *nxf-1(t2160*ts*)* mutant led to activation of RNA transport and mRNA surveillance pathways. *nxf-1* expression itself, its binding partner *nxt-1*/p15 and other genes involved in RNA transport are significantly upregulated when *nxf-1(t2160*ts*)* is mutated. This result likely suggests the existence of a transcriptional feedback mechanism that activates mRNA export in response to low levels of cytoplasmic RNA. Similar transcription-translation feedback loops (TTFL) in which genes are transcribed until their protein products accumulate and are transported into the nucleus, thus inhibiting positive elements from the promoter region of the gene so that transcription is halted, have been described from yeast to mammals [78, 79, 80, 81, 82]. Consistently, genes involved in other aspects of the RNA life cycle such as ribosome biogenesis pathways appear as significantly downregulated, which again suggests a regulatory transcriptional response to adapt the number of ribosomes to the few transcripts available in the cytoplasm (Table 2, S1 Table, S2 Table, S12 Fig).

**Table 2:** Up- and down-regulated pathways determined by KEGG enrichment analysis. Significant pathways have been selected according to their q-value. Genes, within each category, showing significantly altered expression are shown in the right-hand column.

**Upregulated pathways**		
**Description**	**Q Value**	**Genes**
Cel00562 **Inositol phosphate metabolism**	0.020209576	*inos-1*, *piki-1*, *pic-1*, *plc-3*, *ppk-3*
Cel03013 **RNA transport**	0.01812306	*aly*-1, C05C10.2, C44H9.4, *eef*-1A.2, *npp*-14, *npp*-21, *nxf*-1, *nxt*-1, *pab*-2, R186.7, *rae*-1, *smg*-2, Y65B4A.6
Cel03015 **mRNA surveillance pathway**	0.011016861	*aly*-1, C05C10.2, C44H9.4, *nxf*-1, *nxt*-1, *pab*-2, R186.7, *smg*-1, *smg*-2, Y65B4A.6
Cel04020 **Calcium signaling pathway**	0.011016861	*gsa*-1, *itr*-1, *let*-23, *plc*-1, *plc*-3, *ser*-1, *unc*-68, ZC373.4
**Downregulated pathways**		
**Description**	**Q Value**	**Genes**
Cel00010 **Glycolysis/Gluconeogenesis**	2.54e-05	*aldo*-1, *alh*-1, *alh*-5, *alh*-9, *dlat*-1, *dld*-1, *enol*-1, *fbp*-1, *gpd*-2, *gpd*-3, *pgd*-4, *pck*-1, *pck*-2, *pdha*-1, *pdhb*-2, *pgk*-1, R05F9.6, *sodh*-1
Cel00020 **Citrate cycle (TCA Cycle)**	2.54e-05	*cts*-1, *dlat*-1, *dld*-1, *dlst*-1, *idha*-1, *idhg*-2, *mdh*-1, *mdh*-2, *mev*-2, *pck*-1, *pck*-2, *pdha*-1, *pdhb*-1, *pyc*-1, *sucg*-1, *sucl*-2
Cel00071 **Fatty acid degradation**	0.033452235	*acaa*-2, *acdh*-3, *acdh*-8, *acox*-1, *acs*-17, *acs*-2, *acs*-4, *alh*-1, *alh*-9, *aceh*-6, F54C8.1, *kat*-1, *sodh*-1, T02G5.7
Cel00190 **Oxidative phosphorylation**	1.01e-16	*asb*-2, *asg*-1, *asg*-2, *atp*-2, *atp*-5, C16A3.5, C18E9.4, C25H3.9, C33A12.1, *cco*-2, D2030.4, F26E4.6, F29C4.2, F31D4.9, F42G8.10, F44G4.2, F45H10.2, F53F4.10, *gas*-1, H28O16.1, *hpo*-18, *isp*-1, *lpd*-5, *mev*-1, *nduf*-5, *nudf*-7, *nuo*-1, *nuo*-2, *nuo*-3, *nuo*-6, R04F11.2, R07E4.3, R53.4, T02H6.11, T20H4.5, T27E9.2, *tag*-174, *vha*-11, *vha*-13, *vha*-14, W09C5.8, Y51H1A.3, Y54F10AM.5, Y56A3A.19, Y63D3A.7, Y69A2AR.18, Y71H2AM.4, Y71H2AM.5, Y94H6A.8
Cel00260 **Glycine, Serine and Threonine metabolism**	0.033452235	*alh*-9, *cbl*-1, *cbs*-1, *daao*-1, *dld*-1, *gcst*-1, K01C8.1, R12C12.1
Cel00280 **Valine, Leucine and Isoleucine degradation**	0.001649068	*acaa*-2, *acdh*-3, *acdh*-8, *acdh*-9, *alh*-1, *alh*-9, B0250.5, C05C10.3, *dld*-1, *ech*-6, F09F7.4, F54C8.1, *gta*-1, *kat*-1, T02G5.7, *tag*-173
Cel00620 **Pyruvate metabolism**	3.40e-05	*alh*-1, *alh*-9, *dlat*-1, *dld*-1, F32D8.12, *kat*-1, *mdh*-1, *mdh*-2, *pck*-1, *pck*-2, *pdha*-1, *pdhb*-1, *pyc*-1, T02G5.7
Cel00640 **Propanoate metabolism**	0.033452235	*acdh*-8, *alh*-1, *alh*-9, *ach*-6, F09F7.4, *gta*-1, *kat*-1, *sucg*-1, *sucl*-2, T02G5.7
Cel03010 **Ribosome**	1.02e-24	C37A2.7, rla-1, rla-2, *rpl*-1, *rpl*-10, *rpl*13, *rpl*-15, *rpl*-17, *rpl*-18, *rpl*-19, *rpl*-21, *rpl*-22, *rpl*-23, *rpl*-26, *rpl*-27, *rpl*-28, *rpl*-29, *rpl*-3, *rpl*-30, *rpl*-33, *rpl*-34, *rpl*-36, *rpl*-38, *rpl*-39, *rpl*-43, *rpl*-5, *rpl*-6, *rpl*-7A, *rpl*-9, *rps*-1, *rps*-12, *rps*-14, *rps*-15, *rps*-16, *rps*-19, *rps*-2, *rps*-20, *rps*-21, *rps*-23, *rps*-25, *rps*-26, *rps*-28, *rps*-29, *rps*-3, *rps*-30, *rps*-5, *rps*-7, *rps*-8, *rps*-9, *ubl*-1, *ubq*-2, W01D2.1
Cel03050 **Proteasome**	0.000247482	C10G11.8, *dss*-1, F56F11.4, *pas*-3, *pas*-4, *pas*-6, *pas*-7, *pbs*-1, *pbs*-5, *pbs*-6, *pbs*-7, *rpn*-10, *rpn*-3, *rpn*-5, *rpt*-4, *rpt*-6

In addition, a Gene Ontology analysis [77] of the same sets of differentially expressed genes revealed that they do not randomly fall within different molecular function categories. Instead, the significantly upregulated set in the *nxf-1* mutant is highly enriched for GTPase binding, RasGTPase binding, small GTPase binding, actin binding and cytoskeleton protein binding genes. On the other hand, the set of significantly downregulated genes is enriched in genes involved in oxidative phosphorylation and mitochondrial ATP synthesis (Table 2, S12 Fig). These results suggest that reduction of mRNA export has a deep impact on cytoskeletal dynamics that could underlie the *nxf-1*(*t2160*ts) epidermal and mitochondrial defects. These results prompted us to specifically study the cytoskeleton and mitochondrial network in the *nxf-1*(*t2160*ts) mutant.

**Fig 8:**
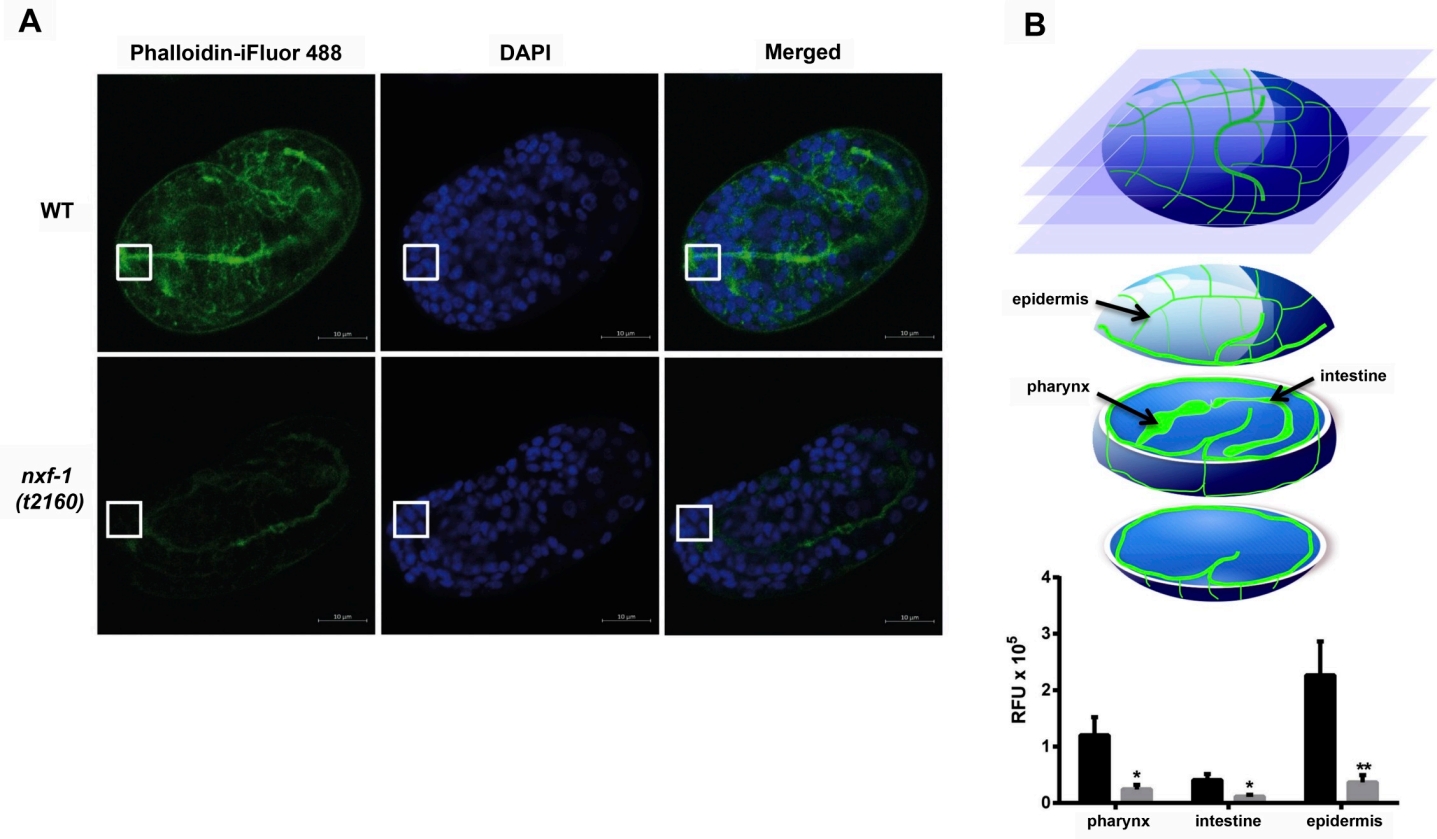
F-actin visualized by Phalloidin-iFluor 488 staining. **(A)** F-actin is apically enriched in arcade cells in WT worms. White squares indicate the anterior end and arcade cells. **(B)** Images of phalloidin stained embryos were taken at different focal planes. Epidermal fluorescence was measured at the upper levels of the embryo whereas pharyngeal and intestinal fluorescence were measured at the center of the embryo. The histogram shows the decrease in F-actin staining in the *nxf-1(t2160*ts*)* mutant compared to the WT (n = 14) on different regions of the embryo (pharynx, intestine and epidermis). F-actin is significantly reduced throughout the *nxf-1(t2160*ts*)* embryos. Student’s t-test; *p<0.05, **p<0.01. n = 20. Scale bar: 10μm.

Cytoskeletal growth and rearrangement require the translation of specific mRNAs that code for structural components and regulatory proteins connected to the cytoskeleton [83]. To examine the actin filament network in WT and *nxf-1*(*t2160*ts) embryos, we used phalloidin staining. Whereas WT embryos accumulated actin at the nascent apical surface at the onset of epithelialization, we observed a decrease in filamentous actin (F-actin) staining in the mutant. In addition, actin remained dispersed in the arcade cells of *nxf-1*(*t2160*ts) embryos compared to WT (Fig 8). Next, we evaluated the mitochondrial network morphology by discriminating between four types of mitochondrial shapes: connected, intermediate, fragmented and very fragmented [84]. *C*. *elegans* WT embryonic cells show a connected mitochondrial network in their cytoplasm (S13 Fig). In contrast, *nxf-1* (*t2160*ts) embryos grown at 25°C showed a general dotted pattern of Mitotracker staining in their cytoplasm, indicating the additional presence of fragmented-type mitochondria (S13 Fig). To further validate this observation, we analyzed mitochondrial morphology in adult muscle cells, a tissue where mitochondria are highly abundant and evident. To do so, animals were grown for 8 days at 25°C and scored at day 1 post L4, day 4 and day 8. 64% (n = 47) of the *nxf-1*(*t2160*ts) body wall muscle cells already showed a fragmented pattern of mitochondrial network at day 1 (S14 Fig). A higher percentage (76%) (n = 46) of *nxf-1*(*t2160*ts) muscle cells still had the fragmented phenotype at day 8, whereas in WT worms, only 14% (n = 21) of muscle cells showed this mitochondrial morphology (S14 Fig). This fragmented mitochondrial network observed in *nxf-1*(*t2160*ts) is detectable in different types of embryonic cells and not restricted to epithelia. Therefore, it does not seem to be the cause of morphogenetic defects but rather a result of cytoskeletal defects [85].

These data, as a whole, point to a model in which the decrease in cytoplasmic mRNA available for actin rearrangement could explain the reduction and disorganization of the actin cytoskeletal network in *nxf-1*(*t2160*ts) mutant embryos, leading to cell attachment and elongation defects [86,87]. The transcriptional activation of genes coding for small GTPase, actin and cytoskeleton binding proteins further supports the existence of a transcriptional feedback mechanism that activates the expression of those genes in response to the cellular requirements of cytoskeletal rearrangements.

### Proteomic analysis defines a molecular model of RNA transport and recycling of RNA transporters

To get deeper insight into the molecular mechanism by which NXF-1 acts in the cell, we identified *C*. *elegans* NXF-1 co-immunoprecipitated protein partners using LC-MS/MS (liquid chromatography-mass spectrometry/mass spectrometry). We expressed NXF-1::3xFLAG::eGFP to immunoprecipitate NXF-1 along with its protein partners and used the N2 WT strain as the negative control. Immunoprecipitations (IPs) from three replicate JCP519 and N2 worm extracts were eluted from the beads by competitive elution with the 3xFLAG peptide. Next, immunoprecipitates were resolved by SDS-PAGE, and stained with Coomassie Blue. Proteins were identified by LC-MS/MS (Table 3). Co-immunoprecipitated proteins fall into the following two main categories:

**Table 3:** NXF-1 and its protein partners co-immunoprecipitated by LC-MS/MS (Liquid Chromatography-Mass Spectrometry/Mass Spectrometry).

Protein	Quantitation/Spectral Counts*						Description
	N2_1	N2_2	N2_3	NXF-1_1	NXF-1_2	NXF-1_3	
**NXF-1**	0	0	0	65	60	63	Nuclear RNA export factor 1
**NXT-1**	0	0	0	9	25	21	NTF2-related export protein
**NPP-9**	0	0	0	93	104	109	Nuclear pore complex protein
**GLH-1**	0	0	0	4	2	5	ATP-dependent RNA helicase
**CAR-1**	0	0	0	5	1	4	Cytokinesis, Apoptosis, RNA-associated
**PAB-1**	0	0	0	2	0	5	Poly(A) binding protein
**CGH-1**	0	0	0	2	0	1	ATP-dependent RNA helicase
**CEY-2**	0	0	0	1	0	2	*C*. *elegans* Y-box
**HRP-2**	0	0	0	1	0	3	human HnRNP A1 homolog
**EEF-1B.2**	0	0	0	2	1	0	Probable elongation factor 1-beta/1-delta 2
**F55A12.5**	0	0	0	10	6	9	Uncharacterized protein
**VIT-1**	0	0	0	52	28	43	Vitellogenin-1
**VIT-2**	0	0	0	69	0	53	Vitellogenin-2
**VIT-4**	0	0	0	0	0	33	Vitellogenin-4
**RAN-1**	1	0	0	14	19	21	GTP-binding nuclear protein
**RAN-2**	0	0	0	3	1	1	Ran GTPase-activating protein 2
**ANC-1**	0	0	1	4	0	1	Nuclear anchorage protein 1
**IMB-3**	0	0	0	0	1	1	Importin beta family
**VIG-1**	0	0	0	3	0	6	VIG (Drosophila Vasa Intronic Gene) ortholog
**FIB-1**	0	0	0	4	0	3	rRNA 2'-O-methyltransferase fibrillarin
**CSQ-1**	0	0	0	0	2	4	Calsequestrin
**CTL-3**	0	0	0	1	0	1	Catalase
**ABTS-3**	0	0	0	6	0	1	Anion/Bicarbonate Transporter family
**F46H5.7**	0	0	0	3	1	0	From *S*. *cerevisiae* homology- plasma membrane protein; involved in G-protein mediated pheromone signaling pathway
**C44E4.4**	0	0	0	1	0	4	Ortholog of human SSB (Sjogren syndrome antigen B)
**HCP-1**	0	0	0	1	0	1	Homolog of the mammalian centromere protein-F (CENP-F)
**HSP-16.1**	0	0	0	1	0	1	16-kD heat shock protein (HSP)
**C01G10.8**	0	0	0	1	0	2	Ortholog of human AHSA1 (activator of Hsp90 ATPase activity 1)

#### Factors involved in mRNA metabolism

As expected, we co-immunoprecipitated the NXF-1 binding partner, NXT-1 [58] and the NCP cytoplasmic fibril component NPP9/RanBP2/NUP358 that is involved in disassembly of the export complex [88] (S15 Fig, Table 3). RanBP2 provides a major binding site for NXF1/TAP at the NPC cytoplasmic filaments, thereby restricting its diffusion into the cytoplasm after NPC translocation. NPP-9 could then act as a physical link between NPCs and P granules for NXF-1-dependent transport [89,90]. In further support of this function, we co-immunoprecipitated the P-granule DEAD box RNA helicase GLH-1. GLH-1 could facilitate the passage of NXF-1 and its cargo from NPCs into perinuclear P-granules before releasing mRNA into the general cytoplasm (S15 Fig, Table 3) [28]. Once in the cytoplasm, mRNA is within large ribo-protein complexes that may locate at the nuclear envelope (germline P granules) or remain isolated within the cytoplasm (processing P bodies; stress granules) [28,91,92,93].

In consonance, we also co-immunoprecipitated P body components: poly(A) binding protein PAB-1/PABP, decapping factor CGH-1/DDX6, CAR-1/Rap55/Trailer hitch and Y-box protein CEY-2 (S15 Fig, Table 3). Interaction between these proteins within the P bodies has been described in *C*. *elegans*, although this interaction seems to be RNA-mediated [94,95,96]. Co-Immunoprecipitation of these proteins, together with detection of NXF-1 in cytoplasmic granules of mature oocytes (Fig 3B, S15 Fig, Table 3), suggests the involvement of NXF-1 in mRNA metabolism after transport, such as storage of maternal product within P bodies or as sites for mRNA degradation.

Our mass spectrometry analysis detected other proteins involved in different aspects of mRNA metabolism, such as alternative splicing factor HRP-2 [97]. However, the low spectral count of these proteins suggests that these interactions might not be direct but rather mediated through the RNA molecules to which they are bound.

#### Proteins involved in other types of transport

We detected importin IMB-3/karyopherin-β3 and GTP-binding proteins RAN-1/Ran GTPase, RAN-2/RanGAP in our IP/Mass spectrometry analysis. IMB-3 is an importin-beta-like protein orthologous to *Drosophila*, vertebrate, and yeast importin/karyopherin-β3. The N-terminal tail of NXF-1/NXF1 contains an NLS (nuclear localization signal), which is recognized by karyopherins at the cytoplasmic site of the NPC favoring its import. Detection of IMB-3 and RAN-1 together with the cellular localization of *C*. *elegans* NXF-1 (Fig 3, Table 3), strongly suggests that after exporting mRNA to the cytoplasm, RNA-free NXF-1 could be recycled back to the nucleus by IMB-3 importin in a Ran-dependent pathway (S15 Fig).

In *C*. *elegans*, asymmetric distribution of the GTP- and GDP-bound conformations of the small GTPase RAN-1 across the nuclear envelope (NE) serves as a gradient in many transport processes [7,90,98]. RAN-2/RanGAP stimulates GTPase activity of RAN-1 which causes cytoplasmic release of the cargo from the exportin complex. Therefore, co-immunoprecipitation of RAN-2 suggests that NXF-1 could export cargo molecules in a RAN-1 GTPase-dependent cycle (S15 Fig, Table 3). Interestingly, in human HeLa cells, NXF1 and karyopherin β2B collaborate in the export of a proportion of mRNAs in RanGTP dependent cycle [99].

## Discussion

The formation and maintenance of specialized organs depend on developmental signaling pathways that regulate cell proliferation and differentiation, as well as establishment of the correct architecture by regulating cell-cell adhesion, cytoskeletal organization and apical-basal polarity within the constituent cells. For this to happen, gene expression has to be tightly regulated in all the steps; from transcription to mRNA export and translation [1,3]. Three levels of regulation control formation of the arcade cell epithelium: first, the transcriptional level; second, the level of protein expression; and third, the protein localization to nascent adherens junctions [36].

Nuclear export factor 1 (NXF-1), but not its ortholog, NXF-2, has been shown to play an essential role in mRNA export in *C*. *elegans* [41,59,60]. However, the consequence of NXF-1 partial loss-of-function was not examined previously. Isolation of the *nxf-1*(*t2160*ts) thermo-sensitive mutant provides an invaluable tool for analyzing the spatial and temporal *in vivo* role of mRNA transport during development. *nxf-1*(*t2160*ts) mutation results in mRNA accumulation in all cell nuclei. This inability to export mRNA primarily disrupts epidermal and pharyngeal morphogenesis during embryonic development. Mutant embryos do not elongate properly and show problems with epidermal cell organization. In addition, although the pharynx was evident and the pharyngeal lumen was visible, in most of the cases (87.5%, n = 176) it was unattached to the mouth.

Our genetic analysis revealed that the observed phenotypes were not the result of cell fate mis-specification, but rather cell morphogenetic defects. Expression of *pha-4*, a transcription factor that regulates pharyngeal development [46,100]; *myo-2*/Myosin-3, pharyngeal muscle myosin [44] and *ric-19*/ICA1, which is expressed in nervous system [45], revealed that the fate of major pharyngeal components was properly specified. In contrast, expression of membrane-tagged GFP [32,56] and the apical junction markers: PAR-6/PARD6A; DLG-1/Discs large [101,102]; AJM-1 [62] and HMR-1/E-cadherin [103] indicated that Pun pharynxes of *nxf-1*(t2160*ts*) animals are possibly a result of lost cell polarity and failed epithelialization of arcade cells. Similar expression patterns of apical markers have been observed in *pha-1* mutants [104].

These developmental defects do not reflect a specific function of NXF-1, but rather the consequence of the reduction of mRNA export. Disruption of other nuclear export factors such as NXT-1/p15 and HEL-1/UAP56 also led to similar embryonic lethality, epidermal defects and the Pun phenotype. Thus, although it affects all cells, hypodermal and especially pharyngeal development seem to be particularly sensitive to a reduction in the efficiency of mRNA export. The arcade cell epithelium forms extremely rapidly, in less than 10 min, while epidermal epithelialization takes over 30 min [32,33]. Therefore, pharyngeal morphogenesis probably requires extremely tight temporal control over the differentiation process.

As a consequence of the low amount of cytoplasmic mRNA, *nxf-1* mutation causes upregulation of genes involved in mRNA export and downregulation of ribosomal RNAs. *aly-1*/ALYREF, *eef-1A*.*2*/EEF1A1, *npp-14*/NUP214, *npp-21*/TPR, *nxt-1*/p15 and *nxf-1* itself, among others, appear significantly upregulated in *nxf-1*(*t2160*ts) worms compared to WT N2 animals (S1 Table, S2 Table). Such a feedback mechanism has also been described in *Drosophila* Schneider cells (S2 cells) in which blocking the NXF1-mediated mRNA export pathway results in upregulation of export factors [105].

A similar feedback regulatory mechanism also seems to operate for genes involved in cytoskeletal rearrangement. *nxf-1* loss of function causes the lack of an apical junction in arcade cells (Fig 5, S3 Fig, S4 Fig) and a dramatic reduction in filamentous actin in *nxf-1* mutant embryos (Fig 8). Our transcriptomic analysis shows a significant upregulation of genes involved in cytoskeletal maintenance: GTPase binding, Ras GTPase binding, small GTPase binding, Rho GTPase binding, actin binding, and cytoskeletal binding proteins. This overexpression likely occurs as a feedback mechanism due to an insufficiency of cytoplasmic mRNAs necessary for cytoskeletal maintenance and rearrangement. Transcriptional activation of these genes is indeed a critical step during epithelial polarization and cytoskeletal reorganization [87].

Studies in *Drosophila* suggest a functional connection between SBR/NXF1 and the cytoskeleton [106]. In early *D*. *melanogaster* embryos, SBR/NXF1 marks the spindles of dividing nuclei [107]. We found the HCP-1/CAGE1 protein among the NXF-1 interactors in the immuno-precipitation experiments. HCP-1 is a centromere-associated protein involved in the fidelity of chromosome segregation [108]. The key role of HCP-1 is to target CLS-2/CLASP to kinetochores which promote the polymerization of kinetochore-bound microtubules [109]. Detection of HCP-1 suggests that NXF-1 may play a role in mitotic spindle assembly independently of mRNA transport. This functional connection between NXF1 and the embryonic mitotic spindle may underlie the slow cell division rate and the DNA breaks observed in *C*. *elegans nxf-1* (*t2160*ts) mutants. *D*. *melanogaster* sbr10 and sbr5 mutants have morphological spindle defects in their first meiotic division [110]. Moreover, the sterile males of sbr12 mutant flies display immobile spermatozoa which exhibit disturbances in mitochondrial morphology and cytokinesis similar to those described here [106,107].

In addition to the defects in epidermal and pharyngeal morphogenesis, *nxf-1* loss of function reduces the gonadal mitotic regions in *C*. *elegans*. The reduced number of mitotic germ cells in *nxf-1*(*t2160*ts) animals and the small number of cells in M-phase could be explained by mitotic delay of cells entering into the M-phase, which leads to mitotic defects and increased CDK-1 phosphorylation levels (Fig 6, S10 Fig). *C*. *elegans* germline proliferation is governed by GLP-1/Notch-receptor and other regulators [69, 70]. Our results suggest that efficient mRNA export of those and/or other factors is key to proper mitotic progression in the *C*. *elegans* gonad. Thus, knockdown of HEL-1/UAP56 also leads to increased CDK-1 phosphorylation levels (S10 Fig). Interestingly, UAP56/HEL-1 associates with the mitotic apparatus in HeLa cells. When UAP56/HEL-1 was knocked down, chromosome misalignment and mitotic delay at prometaphase were frequently observed in mitotic cells. Chromosome misalignment causes activation of the spindle assembly checkpoint (SAC) which arrests mitotic progression at prometaphase [111].

Interestingly, not only mitosis but also meiosis is affected in *nxf-1*(*t2160*ts) animals. The massive accumulation of RAD-51 foci in the meiotic region suggests the existence of multiple DNA breaks. Importantly, knockdown of other mRNA export factors such as HEL-1/UAP56 does not lead to the same accumulation of RAD-51 foci, suggesting that they are not caused by the lack of mRNA export (S11 Fig). These breaks could form as a consequence of the impaired cytoskeleton dynamics during chromosome pairing or could reflect the existence of torsional stress at the DNA fiber level upon NXF-1 downregulation. This mechanical stress activates ATR which seems to modulate nuclear envelope plasticity and to promote chromatin detachment from the nuclear envelope [112,113]. Unexpectedly, high levels of mRNA from a transgene containing the *hsp-16*.*2* promoter, GFP, and the *unc-54* 3’UTR (*hsp-16*.*2*::*gfp*::*unc-54 (3’UTR))*, has been detected in the so-called “expression zone” [26] that overlaps with the region where we see the meiotic RAD-51 accumulation in the *nxf-1*(*t2160*ts) mutant. Additional studies in *C*. *elegans* show that the heat shock *hsp-16*.*2* gene promoter relocates to the nuclear periphery after heat shock [114]. These findings suggest the existence of a yet unknown stress response mechanism in the late pachytene/diplotene germ cells.

In summary, mRNA export is required in all tissues and organs. However epithelial cells that undergo a rapid morphogenetic transformation during development (such as arcade cells and epidermis) and the germline (the only proliferative tissue in adult nematodes) appear to be highly sensitive to reductions in the mRNA export rate in *C*. *elegans*. Many proteins involved in mRNA export have been implicated in cancer, developmental and neural diseases [1,115,116,117,118,119]. It has been shown that NPC can be reprogrammed as part of the oncogenic transformation process, the result of a viral infection or during oxidative and metabolic stress [1,8]. Interestingly, bioinformatic research predicts NXF1 to be a probable tumor suppressor gene (TSG) [120]. A deeper understanding of the processes involved in mRNA export from nucleus to cytoplasm is required. Basic aspects of their relationship to stress and DNA damage response remain an open question. This knowledge will shed light on many aspects of biology ranging from cell differentiation to morphogenesis and disease.

## Materials and methods

### *C*. *elegans* strains and maintenance

Standard methods were used to culture and manipulate *C*. *elegans* strains [121]. Worms were grown on NGM (nematode growth media) agar plates. Plates were previously seeded with an LB (Luria-Bertani) liquid culture of the *Escherichia coli* strain OP50 (Uracil auxotroph, *E*. *coli* B., ampicillin resistant from CGC) overnight at 37°C (ampicillin (100 mg/ml) and nystatin (0.004%)), and air-dried.

When larger amounts of worms were needed (for IP experiments), egg-seeded plates were used. Egg plates were prepared as described [122]. Normal NGM plates were seeded with 5ml egg mix and air-dried.

In this study, worms were grown at 15°C and 25°C. The *nxf-1(t2160*ts*)* mutant is temperature sensitive so it was maintained at 15°C. Before all experiments, worms were shifted to the non-permissive temperature of 25°C. The *C*. *elegans* strains used in this study are listed in S3 Table. Their genotypes, characteristics and sources are shown.

### 3D Fluorescence in situ hybridization (3D-FISH)

3D FISH protocol [123] was followed. Embryos were fixed on slides using the freeze-crack procedure. For hybridization, the probe against the poly-A sequence of mRNA (40T) labeled with Cy3 fluorochrome (Sigma) was added to the hybridization buffer and slides were incubated for 2–3 days at 37°C.

### Immunostaining

Phalloidin staining [124] was performed. Embryos were fixed on slides using the freeze-crack procedure. After cracking, eggs were fixed for 20 minutes in fix/permeabilization solution (4% PFA; 0.2% Triton X-100; 50mM PIPES pH 6.8; 25mM HEPES pH 6.8; 10.2mM EGTA; 2mM MgCl_2_), then slides were rehydrated/permeabilized by three 5-minute washes in 1X PBS in a Coplin jar, followed by 90 minutes of incubation with CytoPainter Phallooidin-iFluor 488 solution (Abcam). Slides were washed 2 times in 1X PBS and mounted by adding a drop of ProLong^TM^ Diamond Antifade Mountant with DAPI (Invitrogen).

Young adult worms were dissected in dissection buffer (1X egg buffer, 0.02% Tween-20, 0.2mM Levamisole and Milli-Q H_2_O). Dissected gonads on slides were fixed in fixation buffer (1X egg buffer, 0.02% Tween-20, 4% formaldehyde and Milli-Q H_2_O) covered with a coverslip (24x24 mm), incubated for 5 minutes and dipped in liquid nitrogen. Coverslips were flipped away and slides were incubated in Coplin jars in precooled (-20°C) 1:1 acetone: methanol solution for 10 minutes. Next, slides were washed three times (10 minutes each) in 1% Triton PBS buffer followed by another 5-minute wash with 0.1% Tween-20 PBS. Samples were blocked for 20–30 minutes in a Coplin jar with 10% fetal bovine serum diluted in 0.1% Tween PBS. Slides were pre-blocked for 20–30 minutes using Image- iT FX Signal Enhancer (Invitrogen). Slides were incubated with the desired first antibody, washed three times (10 minutes each) in 1% Triton PBS buffer, stained with the appropriate secondary antibody, and mounted by adding a drop of ProLong^TM^ Diamond Antifade Mountant with DAPI (Invitrogen). The following antibodies were used: anti-RAD-51 (1:10000, SDIX 2948.00.02); anti-pH3 (detects pSer 10 H3, 1:400, Santa Cruz Biotechnology sc-8656R); anti-pTyr15 CDK-1 (1:10000, CALBIOCHEM 213940); goat anti-rabbit IgG (H+L), Alexa Fluor 555 (1:1000, Thermo Fisher Scientific); goat anti-rabbit IgG (H+L), Alexa Fluor 488 (1:1000, Thermo Fisher Scientific).

### Immunoprecipitation from *C*. *elegans* extracts

In order to immunoprecipitate NXF-1 and Co-IP their interactors, protein extracts from JCP519 (*nxf-1(t2160*ts*) V; jcpEx6[pAZ09(*P*nxf-1*::*nxf-1*::*3xFLAG*::*eGFP*::*nxf-1UTR)])*, were used. Extracts from WT worms were used as the negative control. A large amount of protein extract was needed, and thus 8 to 10 NGM egg plates were used. Protein extracts were measured using the *BCA Protein Assay Kit* (Fisher Scientific) according to the manufacturer’s instructions. IP/Co-IPs were performed with *Anti-FLAG M2 Magnetic Beads* (Sigma) composed of the murine derived ANTI-FLAG M2 monoclonal antibodies attached to superparamagnetic iron impregnated 4% agarose beads. The eluted IPs were run on *Mini-PROTEAN TGX Precast Gels* by sodium dodecyl sulfate-polyacrylamide gel electrophoresis (SDS-PAGE) and proteins were separated according to their molecular weights [125]. Next, the gels were stained with Coomassie Blue and bands were excised. Proteomic analysis was performed at the CIC Biogune proteomics platform (https://www.cicbiogune.es/org/plataformas/Proteomics).

### Worm transformations

*C*. *elegans* biolistic bombardment was performed as described [121], with few modifications. We used the *Biolistic PDS-1000/He Particle Delivery System* (Bio-Rad). This system uses high-pressure helium, released by a rupture disk and a partial vacuum, to propel a macrocarrier sheet with millions of microscopic DNA-coated gold particles toward target worms at a high velocity. In this work, JCP495 (*nxf-1(t2160*ts*)* V) was successfully rescued by bombardment with plasmids pAZ07 (P*nxf-1*::*nxf-1*::*nxf-1*UTR) and pAZ09 (P*nxf-1*::*nxf-1*::3xFLAG::eGFP::*nxf-1*UTR) (S1 Fig). The JCP519 (*nxf-1(t2160*ts*)* V; jcpEx6[pAZ09(P*nxf-1*::*nxf-1*::3xFLAG::eGFP::*nxf-1*UTR)]) strain expressing NXF-1::3XFLAG::eGFP was generated by gene bombardment using the plasmid pAZ09.

### RNA interference

In this study, RNAi was achieved by feeding worms with the bacteria that produced the desired dsRNA. RNAi clones of *nxf-1*, *hel-1* and *rnp-4* (our lab), as well as *nxt-1* and *mag-1* [126] were used in this study. The empty L4440 vector in HT115 cells was used as a control. For a “mild” RNAi effect, bacterial RNAi clones of *nxf-1* and *nxt-1* were diluted with L4440 at a 1:1 concentration.

### Microscopy

For microscope preparations, worms were monitored on NGM plates under a Leica Stereo microscope (*MZ16FA*). DIC was performed on a fluorescent Leica microscope (*DM600B*) equipped with a *Hamamatsu Orca-ER C10600* camera fitted with DIC optics. *C*. *elegans* embryos, larvae and adults were mounted on 4.5% agar pads and observed under DIC optics [127]. Images were captured with Micro-manager software (https://micro-manager.org/) and processed with XnView software and ImajeJ or Fiji software.

Confocal microscopy imaging was performed with a Zeiss 780 confocal microscope (immunofluorescence and phalloidin staining experiments). Images were acquired and processed using ZEN lite open software from Zeiss and ImageJ/Fiji.

Relative fluorescence image data obtained from ImageJ/Fiji was statistically analyzed with IBM SPSS Statistic 21, and the representative graphs were created with GraphPad Prism 6 software.

### Next generation sequencing and Whole-genome sequencing (WGS)

In this study, the *nxf-1(t2160*ts*)* strain was backcrossed with the Hawaiian (CB4856) strain. Around 3000 F2 recombinants (*t2160*ts)/(Hawaiian-CB4856) were singled out. 560 thermo-sensitive F2 *t2160*ts/Hawaiian recombinants were obtained. Total DNA extraction of 560 *C*. *elegans* worms (560 recombinants (*t2160*ts*)*/(Hawaiian-CB4856)) was performed using the *Plant/Fungi DNA Isolation Kit* (Norgen Biotek Corp.) following the manufacturer’s instructions. This kit enabled us to isolate total DNA from a small number of worms.

Using the Hawaiian single-nucleotide polymorphism (SNP) mapping method, we backcrossed the *nxf-1(t2160*ts*)* mutant with the polymorphic Hawaiian strain [128]. Next, we isolated the newly generated F2 recombinants homozygous for the *nxf-1(t2160*ts*)* mutation, (Hawaiian-CB4856)/*nxf-1(t2160*ts*)*. Using 205 ng genomic DNA obtained as described, sequencing libraries were constructed using the *NEXTflex Rapid DNA*-*Seq* Kit according to the manufacturer’s instructions (Bioo Scientific). DNA quality and integrity were evaluated by *Experion Automated Electrophoresis System* (Bio-Rad) and the concentration was calculated using qPCR. Libraries were prepared at the genomic platform of the CIBIR (http://cibir.es/es/plataformas-tecnologicas-y-servicios/genomica-y-bioinformatica) and sequenced on an Illumina HiSeq 15000. The quality of DNAseq results was assessed using FastQC(http://www.bioinformatics.babraham.ac.uk/projects/fastqc/). Paired-end 100-bp sequencing yielded a theoretical mean coverage of 245X of the *C*. *elegans* genome. The FastQ files were analyzed using a Cloud-Based Pipeline for Analysis of Mutant Genome Sequences (Cloudmap tool, https://usegalaxy.org/u/gm2123/p/cloudmap) with standard parameters following Cloudmap workflow [42].

### RNA deep sequencing

Total RNA extraction from *C*. *elegans* worms was performed using the *RNeasy Mini Kit* (Qiagen) following the manufacturer’s instructions. Four 99x16.2 mm worm plates of *nxf-1(t2160*ts*)* and WT worms were used.

RNA deep sequencing was performed at the genomic platform of the CIBIR (http://cibir.es/es/plataformas-tecnologicas-y-servicios/genomica-y-bioinformatica). Expression analysis was performed by DESeq2 [129] and edgeR [130] as described [131, 132].

## Supporting information

S1 Fig Schematic drawings of trangenes used in this study. **(A)** plasmid pAZ07 (used to rescue JCP495) and **(B)** plasmid pAZ09 (used to generate transgenic strain JCP519) are shown.
(TIF)

S2 Fig Temperature sensitive curve of *nxf-1*(*t2160*ts) mutant. X axis shows the different developmental stages at which the embryos were temperature-shifted. Y axis shows the percentage of embryonic lethality measured as non-hatched embryos.
Blue line shows the lethality of embryos that underwent an upshift from 15°C to 25°C at the two-cell stage (100% lethality), four-cell stage (73% lethality), twelve-cell stage (73% lethality), 100-cell stage (63% lethality), 200-cell stage (58% lethality), 1.5-fold stage (13% lethality) and 2-fold stage (0% lethality). The lethality does not fall under 50% until mid-embryogenesis, when most cell divisions and epithelialization are completed. Orange line shows the lethality of embryos that underwent a downshift from 25°C to 15°C at the same stages.
Together, the results indicate that mRNA transport is required throughout development but is has to function very efficiently during the morphogenetic events that happen between the 200-cell stage and the 1.5-fold stage.
(TIF)

S3 Fig NXF-1 is essential for arcade cells to form a polarized epithelium. **(A)** Schematic representation of an epithelial cell of *C*. *elegans*. The epithelial cell contains a single junction, the *C*. *elegans* apical junction (CeAJ) with a bipartite organization. The basal region is associated with an electron dense structure, where DLG-1/Disc large and AJM-1 co-localize while the more apical region is not associated with any structure and harbors the HMR-1/E-cadherin and HMP-1/alpha-catenin molecules. **(B-D)** Representative DIC and fluorescence images of 1.5-fold embryos and quantification of expression changes in the *nxf-1(t2160*ts*)* and WT background of the AJM-1::GFP **(B)** and cadherin-catenin complex components HMR-1::GFP **(C)** and HMP-1::GFP **(D)** in pharynx and epidermis. Anterior is to the left. n > 20 embryos for each strain. Statistical analysis was performed using Student’s t-test; *p<0.05, **p<0.01, ***p<0.001, ****p<0.0001.
(TIF)

S4 Fig Apical junction components expression in pharynx and epidermis. **(A-C)** Representative DIC and fluorescence images of 1.5-fold embryos and quantification of expression changes in the *nxf-1(t2160*ts*)* and WT background of the DLG-1::dsRed **(A)** SAX-7::GFP **(B)** and JAC-1::GFP **(C)** in pharynx and epidermis. Anterior is to the left. n > 20 embryos for each strain. Student’s t-test; ***P<0.0001.
(TIF)

S5 Fig Quantification of total levels of PAR-6 in *nxf-1*(*t2160*ts) mutant embryos versus WT embryos. PAR-6 protein is measured as relative fluorescent units (RFU) in *nxf-1*(*t2160*ts) and WT embryos expressing a PAR-6::mCherry transgene (as shown in Fig 4F). The slight increase of PAR-6 protein in the *nxf-1*(*t2160*ts) background suggests that polarization defects in arcade cells are mainly due to mislocalization of PAR-6 and failure in apical localization.
(TIF)

S6 Fig *nxf-1*(*t2160*ts) mutants display a wider intestinal lumen than WT. **(A)** Comparison of WT and *nxf-1*(*t2160*ts) embryonic intestines visualized with DIC and AJM-1::GFP. **(B)** Hatched *nxf-1*(*t2160*ts) L1 larvae show a wider intestinal lumen than WT L1 larvae. **(C)** Quantification of the lumen width of different intestinal sections in WT and *nxf-1*(*t2160*ts) animals. Statistical analysis performed using Student’s t-test; *p<0.05, **p<0.01.
(TIF)

S7 Fig Knockdown of *C*. *elegans* mRNA export machinery members NXF-1/TAP, NXT-1/p15, HEL-1/UAP56. ST65 (ncIs13[*ajm-1*::GFP]) worms in L1 stage were fed bacterial L4440 vector control (a) and RNAi clones of *nxf-1* (b), *hel-1* (c) and *nxt-1* (d). Worms were grown at 15°C and images were taken at the sixth day (adult stage). The worms fed bacterial RNAi clones of *nxf-1* (b) and *hel-1* (c) arrested at the L1-L2 stage whereas worms fed the bacterial RNAi clone of *nxt-1*(d) reached the adult stage and as zoomed in (e), 50% of scored animals show a protruding vulva phenotype (arrows) (n = 260).
(TIF)

S8 Fig DESeq2 normalized expression values and standard deviation for *ego-1*, *ego-2*, *glp-1*, *lag-1*, *lgx-1*, *teg-1* and *teg-4* genes. No significant differences between *nxf-1*(*t2160*ts) samples and WT controls, calculated both by Tukey’s honestly significant difference test (Tukey’s HSD) and pairwise.t.test (FDR ≤ 0.05), are detected.
(TIF)

S9 Fig DESeq2 normalized expression values and standard deviation for *cdc-25*, *cdk-*, histone (*his-*) and *rad-51* genes. Significant differences between *nxf-1*(*t2160*ts) samples and WT controls, calculated both by Tukey’s honestly significant difference test (Tukey’s HSD) and pairwise.t.test (FDR ≤ 0.05), are shown with asterisks. Analyzed genes: *cdc-25*.*1*, *cdc-25*.*2*, *cdc-25*.*3*, *cdc-25*.*4*, *cdk-1*, *his-1*, *his-2*, *his-3*, *his-4*, *his-5*, *his-6*, *his-7*, *his-8*, *his-11*, *his-16*, *his-17*, *his-18*, *his-24*, *his-25*, *his-31*, *his-32*, *his-35*, *his-37*, *his-38*, *his-39*, *his-40*, *his-41*, *his-42*, *his-45*, *his-47*, *his-48*, *his-55*, *his-57*, *his-58*, *his-59*, *his-60*, *his-61*, *his-62*, *his-63*, *his-64*, *his-65*, *his-66*, *his-67*, *his-68*, *his-69*, *his-70*, *his-71*, *his-72*, *his-73*, *his-74*, *rad-51*.
Other histone coding genes such as: *his-9*, *-10*, *-12*, *-13*, *-14*, *-15*, *-19*, *-20*, *-21*, *-22*, *-23*, *-26*, *-27*, *-28*, *-29*, *-30*, *-33*, *-34*, *-36*, *-43*, *-44*, *-46*, *-49*, *-50*, *-51*, *-52*, *-53*, *-54*, *-56* showed no-expression in lab growth conditions.
(TIF)

S10 Fig NXF-1 and HEL-1 are necessary for normal cell proliferation. Mitotic cells in *nxf-1(t2160*ts*)* failed to proceed into mitosis and arrested at G2 phase (**g, h** and **i**), similar to gonads after IR (**d, e** and **f**) and depletion of *hel-1* (**m, n** and **o**). N2 (WT) was used as the negative control (**a, b** and **c**). N2 (WT) and *nxf-1(t2160*ts*)* worms were synchronized. At the L4 stage, part of the N2 worms were fed the *hel-1* bacterial RNAi and the bacterial RNAi clone of the empty L4440 vector was used as a control and fed to the rest of the worms (**j, k** and **l**). Another batch of L4 stage N2 worms were irradiated (90Gy). After 24 hours, gonads of *nxf-1(t2160*ts*)*, N2 irradiated and non-irradiated worms, along with worms fed *hel-1* and L4440, were dissected, fixed, immunostained with α-pTyr-15 CDK-1 and counterstained with DAPI. Scale bar: 10 μm.
(TIF)

S11 Fig Accumulation of RAD-51 in the late pachytene/diplotene region of *nxf-1(t2160*ts*)*. N2 (WT) and *nxf-1(t2160*ts*)* worms were synchronized. At the L4 stage, N2 (WT) worms were irradiated (90Gy). After 24 hours, gonads of N2 non-irradiated (**a, b** and **c**), N2 irradiated (**d, e** and **f**) and *nxf-1(t2160*ts*)* (**g, h** and **i**) worms were dissected, fixed, immunostained with α-RAD-51 and counterstained with DAPI. In another set of experiments, N2 (WT) and *nxf-1(t2160*ts*)* worms were synchronized and from the L1 stage, they were fed the *spo-11* bacterial RNAi (**p, q, r, v, w** and **x**) and the empty L4440 vector (**j, k, I, s, t** and **u**) that was used as a control. At the L4 stage, a fraction of N2 (WT) worms were fed the *hel-1* bacterial RNAi clones (**m, n** and **o**). One-day-old worms were dissected, and their gonads were fixed, immunostained with α-RAD-51 and counterstained with DAPI. Scale bar: 20μm.
(TIF)

S12 Fig Transcriptomic analysis. Statistical analysis with DeSeq and Edger shows 1117 downregulated **(A)** and 834 upregulated genes **(B)** in *nxf-1(t2160*ts*)* vs WT. Gene ontology (GO) analysis, cellular component (CC) analysis, and molecular function (MF) of differentially expressed downregulated and upregulated genes in *nxf-1(t2160*ts*)* vs. WT (**C, D, E** and **F**). The number of genes within each category is represented in color bars, one bar per GO term. Bar length indicates the number of genes belonging to the different GO categories and color indicates the statistical significance, from those with highly significant expression differences (red) to those with low expression differences (blue).
(TIF)

S13 Fig *nxf-1*(*t2160*ts) embryos show a general pattern of fragmented mitochondrial morphology. **(A)** WT embryos at different developmental stages (1–3) show a connected mitochondrial network in the cytoplasm of their cells visualized with Mitotracker staining (1'-3'). **(B)** *nxf-1*(*t2160*ts) mutant embryos at the same developmental stages (4–6) show a general dotted pattern of Mitotracker staining in the cytoplasm of their cells (4’-6’), indicating the additional presence of fragmented-type mitochondria. **(C)** shows a non-stained embryo as a control for autofluorescence (7–7’).
(TIF)

S14 Fig Age-dependent mitochondrial changes occur faster in the *nxf-1(t2160*ts*)* mutant compared to WT. **(A)** Representative images of the different mitochondrial morphologies scored. **(B)** Transgenic animals expressing mitoGFP (ccIs4251 [(pSAK2) P*myo-3*::GFP::LacZ::NLS + (pSAK4) P*myo-3*::mitochondrial GFP + *dpy-20*(+)]) in body wall muscle cells (DAY 1, DAY 4 and DAY 8) were analyzed at different days after the L4 larval stage, respectively (DAY 1 WT n = 41 and *nxf-1(t2160*ts*)* n = 47; DAY 4 WT n = 61 and *nxf-1(t2160*ts*)* n = 73; DAY 8 WT n = 21 and *nxf-1(t2160*ts*)* n = 46). Because *nxf-1(t2160*ts*)* are ts, worms were grown at 15°C and then moved to 25°C at the L4 larval stage. Scale bar: 10μm.
(TIF)

S15 Fig Working model for mRNA export and a new proposed model for the collaboration of NXF-1-RAN-1 in the export of unknown cargo. On the left-hand side, the main steps of mRNA export are shown: NXF-1/NXT-1 recruitment to the mRNP; mRNP export through the NPC where cytoplasmic fibril NPP-9 probably mediates the translocation step of mRNA across the NPC. GLH-1 probably mediates the release of mRNA from P granules into the cytoplasm; mRNAs are stored in P body particles. P body poly(A) binding protein PAB-1, decapping factor CGH-1, CAR-1/Rap55/Trailer hitch and Y-box protein CEY-2 are shown. Finally, import of NXF-1 from the cytoplasm to the nucleus via the IMB-3 transport in the RanGTPase-dependent pathway. On the right-hand side of the proposed model, NXF-1 exports unknown cargo using the RAN-1-dependent pathway. Once in the cytoplasm, RAN-2 associates with the cargo complex and this leads to its dissociation.
(TIF)

S1 Table KEGG Pathway analysis. (DOCX)

S2 Table Genes involved in the RNA transport and mRNA surveillance pathways, upregulated in *nxf-1(t2160*ts*)* compared to WT. (DOCX)

S3 Table *C. elegans* mutant and transgenic strains used in this study and some of their characteristics. (DOCX)

S1 Movie NXF-1::GFP localization throughout the cell cycle during early *C*. *elegans* development. (MOV)

S2 Movie Development of a WT embryo (left) compared to an *nxf-1* (*t2160*ts) embryo (right).
(MOV)
