# Supplementary figures and images for: Reduction of mRNA export unmasks different tissue sensitivities to low mRNA levels during Caenorhabditis elegans development
Source: PLoS Genet. 2019 Sep 16;15(9):e1008338. doi: 10.1371/journal.pgen.1008338 (PMC6762213; doi:10.1371/journal.pgen.1008338)

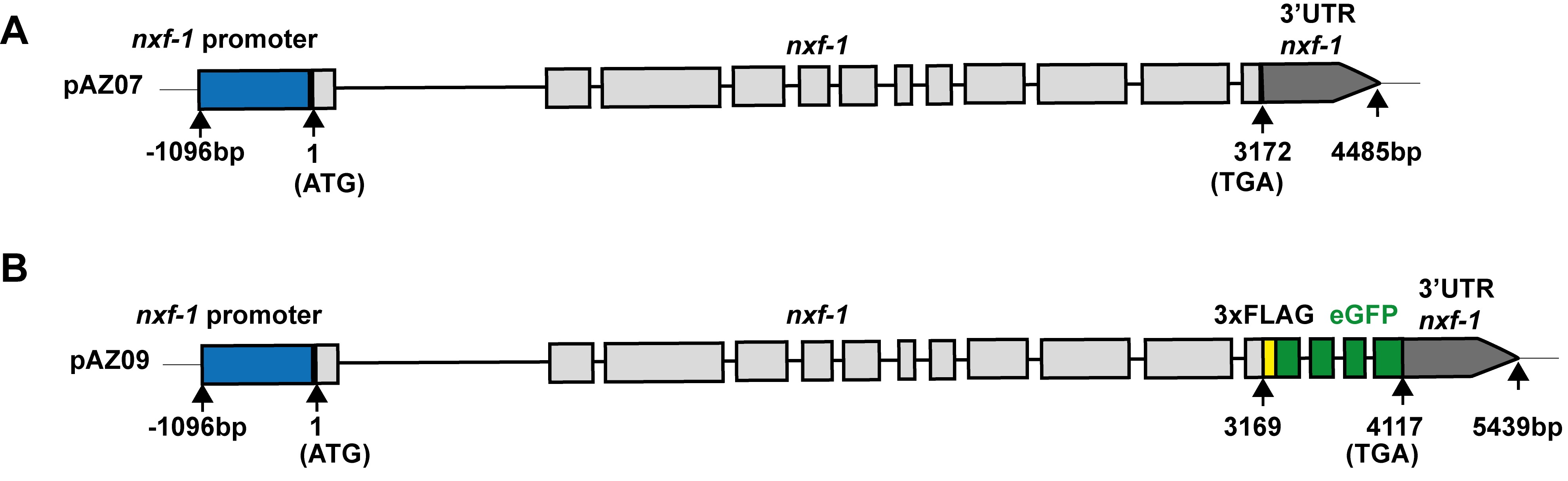

Supplement: S1 Fig — (A) plasmid pAZ07 (used to rescue JCP495) and (B) plasmid pAZ09 (used to generate transgenic strain JCP519) are shown. (TIF) [file pgen.1008338.s001.tif]

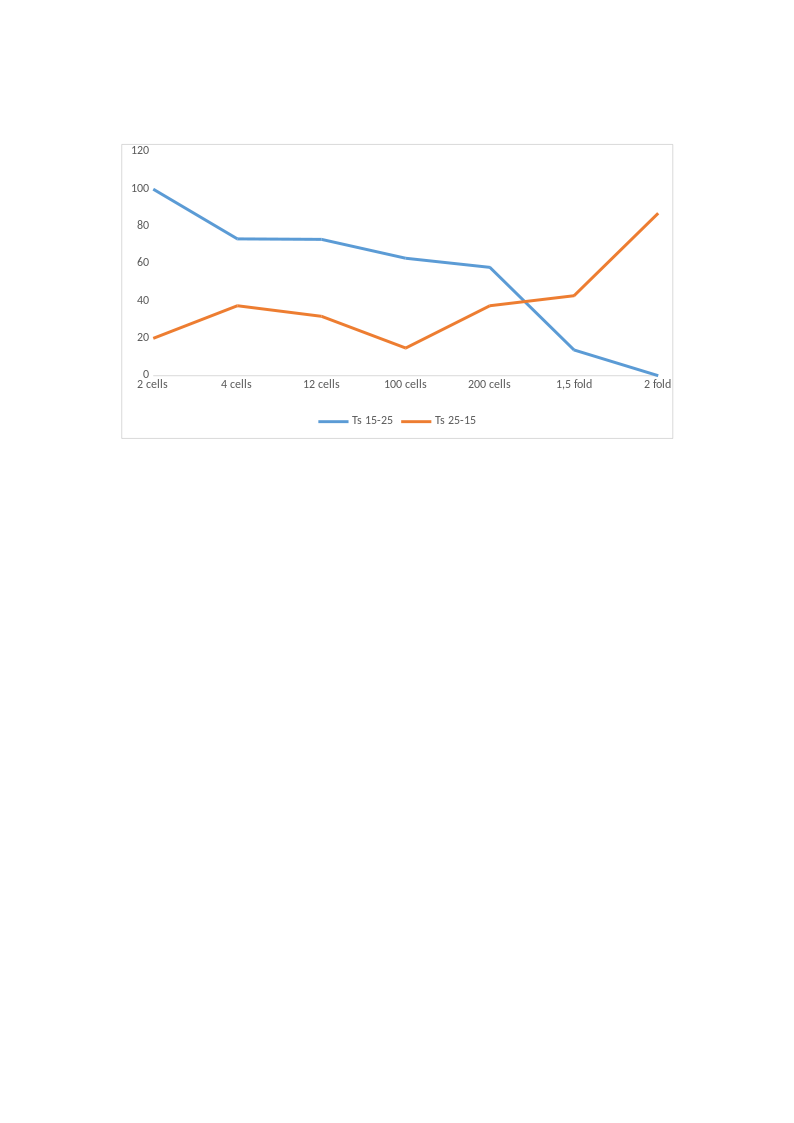

Supplement: S2 Fig — X axis shows the different developmental stages at which the embryos were temperature-shifted. Y axis shows the percentage of embryonic lethality measured as non-hatched embryos. Blue line shows the lethality of embryos that underwent an upshift from 15°C to 25°C at the two-cell stage (100% lethality), four-cell stage (73% lethality), twelve-cell stage (73% lethality), 100-cell stage (63% lethality), 200-cell stage (58% lethality), 1.5-fold stage (13% lethality) and 2-fold stage (0% lethality). The lethality does not fall under 50% until mid-embryogenesis, when most cell divisions and epithelialization are completed. Orange line shows the lethality of embryos that underwent a downshift from 25°C to 15°C at the same stages. Together, the results indicate that mRNA transport is required throughout development but is has to function very efficiently during the morphogenetic events that happen between the 200-cell stage and the 1.5-fold stage. (TIF) [file pgen.1008338.s002.tif]

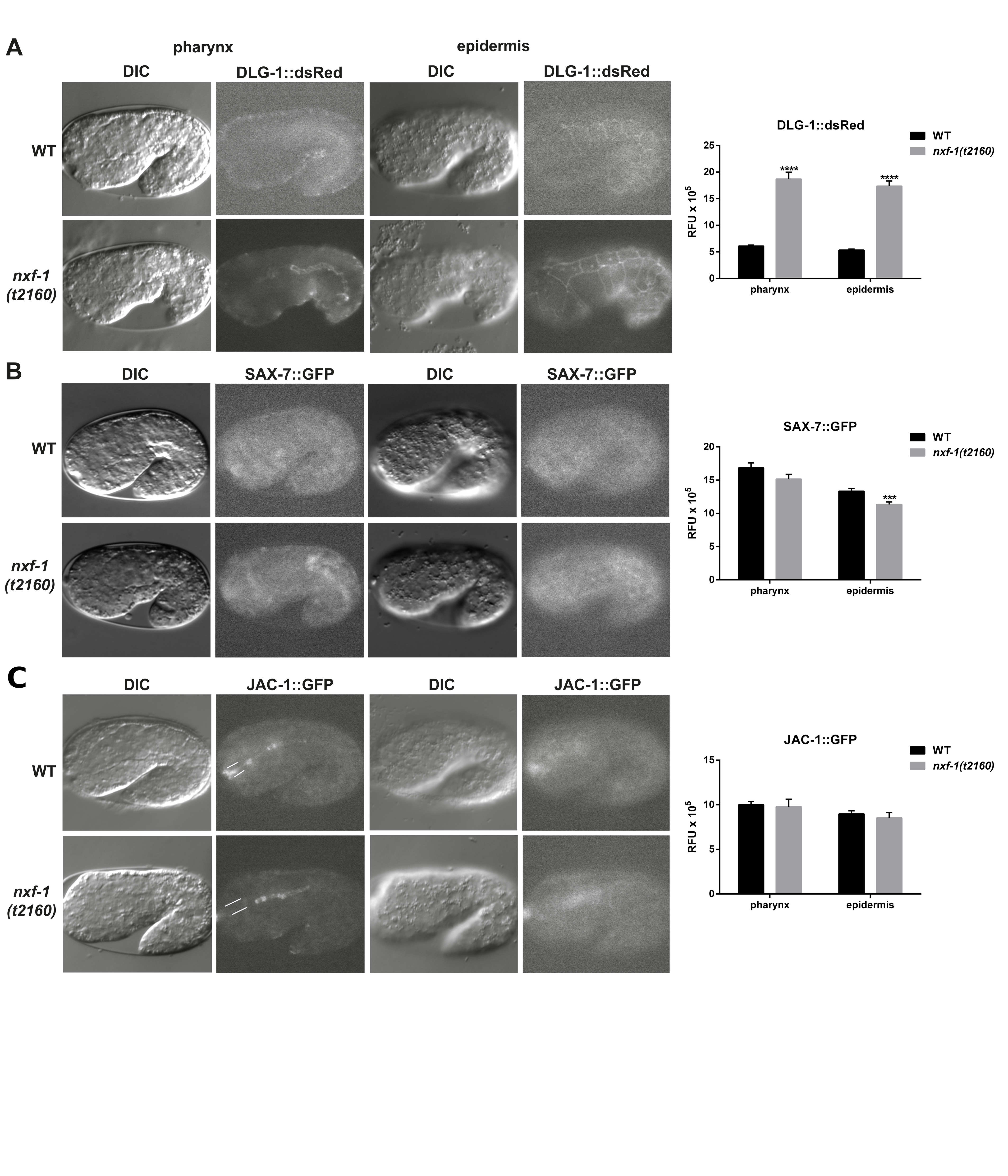

Supplement: S4 Fig — (A-C) Representative DIC and fluorescence images of 1.5-fold embryos and quantification of expression changes in the nxf-1(t2160ts) and WT background of the DLG-1::dsRed (A) SAX-7::GFP (B) and JAC-1::GFP (C) in pharynx and epidermis. Anterior is to the left. n > 20 embryos for each strain. Student’s t-test; ***P<0.0001. (TIF) [file pgen.1008338.s004.tif]

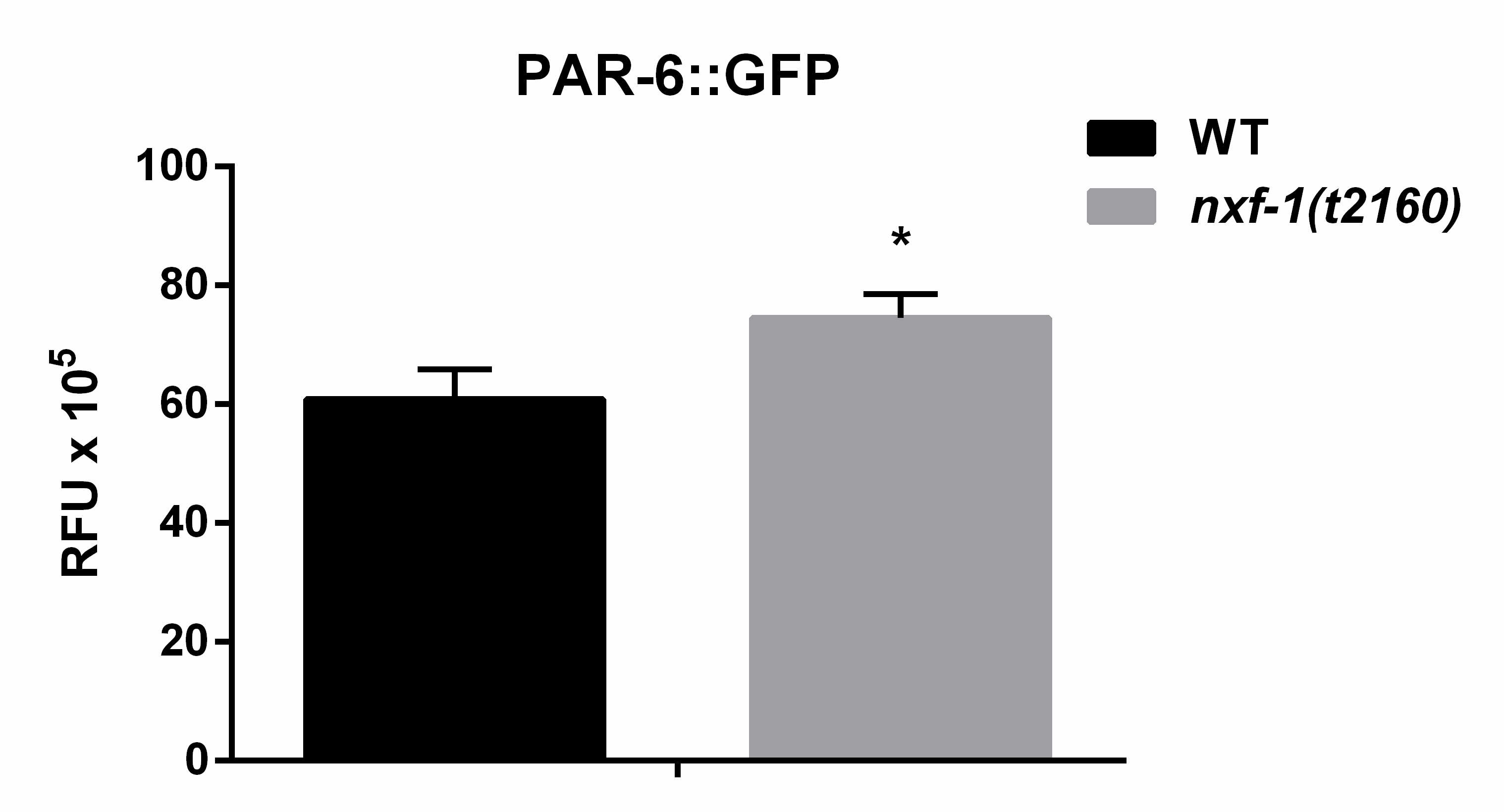

Supplement: S5 Fig — PAR-6 protein is measured as relative fluorescent units (RFU) in nxf-1(t2160ts) and WT embryos expressing a PAR-6::mCherry transgene (as shown in Fig 4F). The slight increase of PAR-6 protein in the nxf-1(t2160ts) background suggests that polarization defects in arcade cells are mainly due to mislocalization of PAR-6 and failure in apical localization. (TIF) [file pgen.1008338.s005.tif]

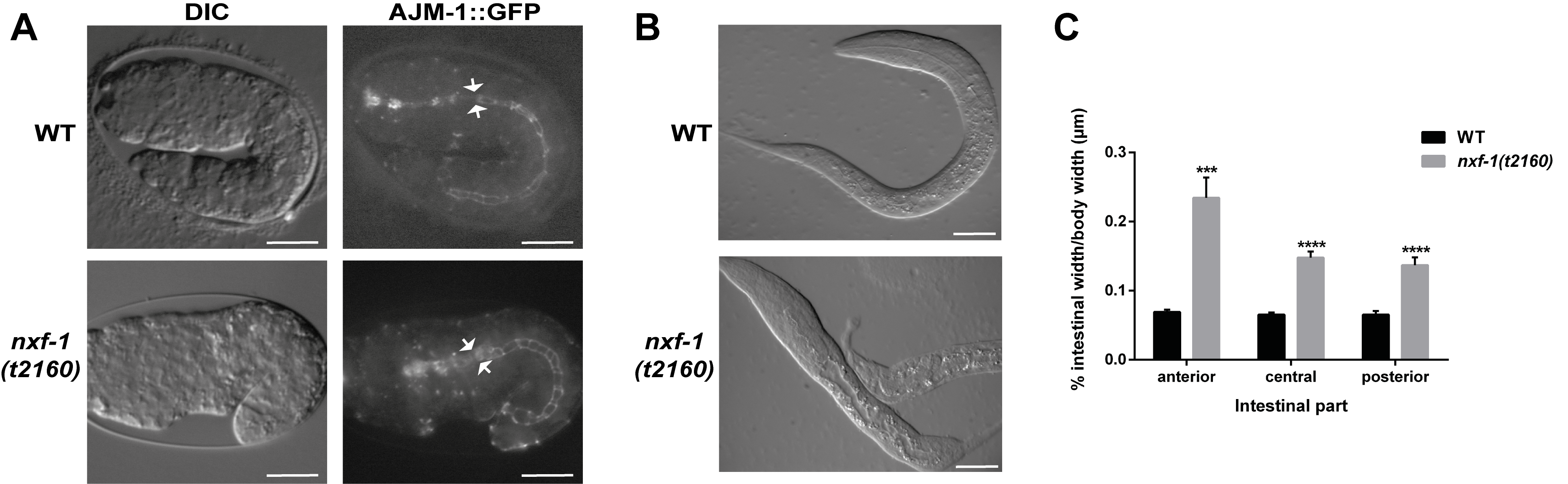

Supplement: S6 Fig — (A) Comparison of WT and nxf-1(t2160ts) embryonic intestines visualized with DIC and AJM-1::GFP. (B) Hatched nxf-1(t2160ts) L1 larvae show a wider intestinal lumen than WT L1 larvae. (C) Quantification of the lumen width of different intestinal sections in WT and nxf-1(t2160ts) animals. Statistical analysis performed using Student’s t-test; *p<0.05, **p<0.01. (TIF) [file pgen.1008338.s006.tif]

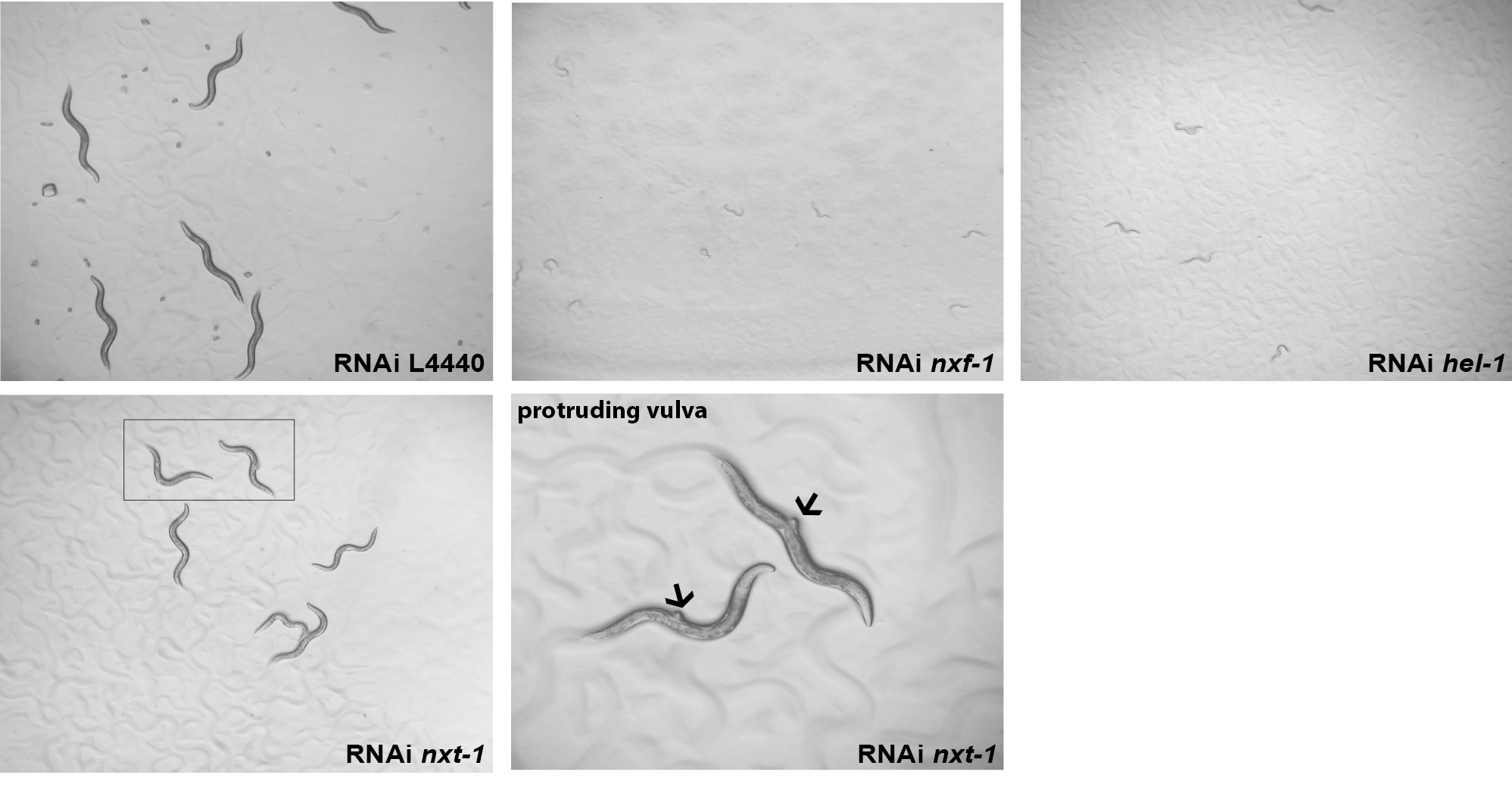

Supplement: S7 Fig — ST65 (ncIs13[ajm-1::GFP]) worms in L1 stage were fed bacterial L4440 vector control (a) and RNAi clones of nxf-1 (b), hel-1 (c) and nxt-1 (d). Worms were grown at 15°C and images were taken at the sixth day (adult stage). The worms fed bacterial RNAi clones of nxf-1 (b) and hel-1 (c) arrested at the L1-L2 stage whereas worms fed the bacterial RNAi clone of nxt-1(d) reached the adult stage and as zoomed in (e), 50% of scored animals show a protruding vulva phenotype (arrows) (n = 260). (TIF) [file pgen.1008338.s007.tif]

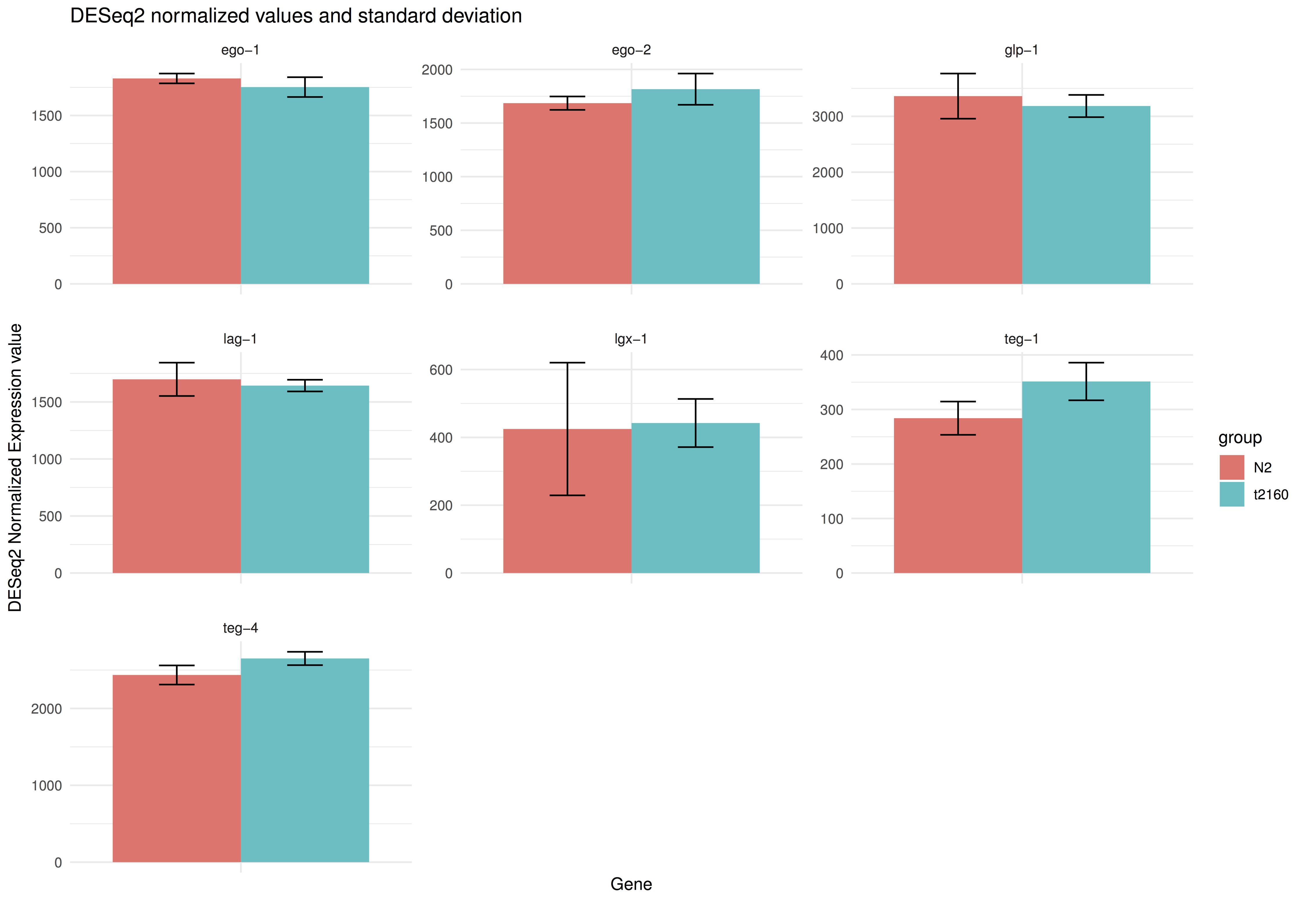

Supplement: S8 Fig — No significant differences between nxf-1(t2160ts) samples and WT controls, calculated both by Tukey’s honestly significant difference test (Tukey’s HSD) and pairwise.t.test (FDR ≤ 0.05), are detected. (TIF) [file pgen.1008338.s008.tif]

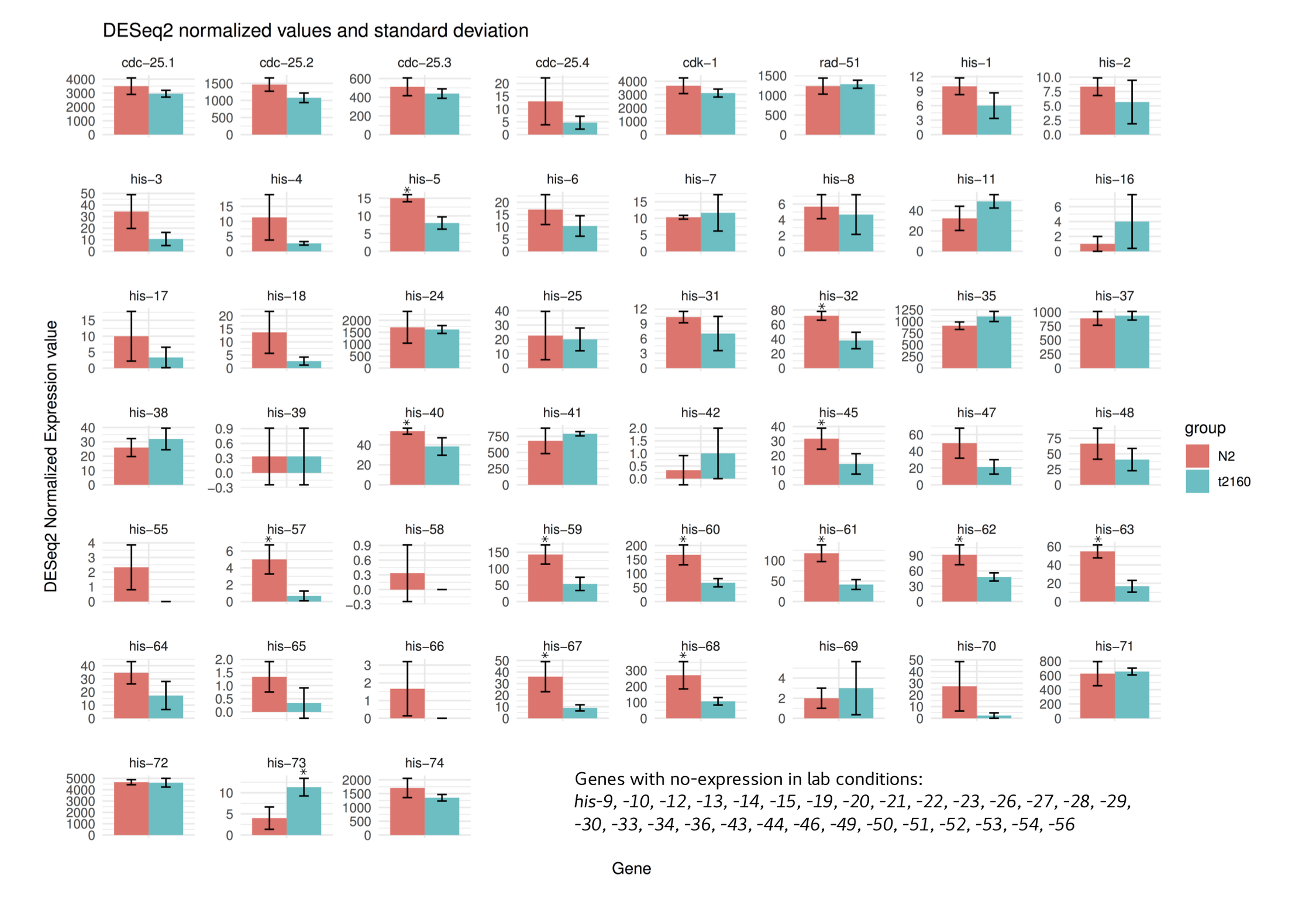

Supplement: S9 Fig — Significant differences between nxf-1(t2160ts) samples and WT controls, calculated both by Tukey’s honestly significant difference test (Tukey’s HSD) and pairwise.t.test (FDR ≤ 0.05), are shown with asterisks. Analyzed genes: cdc-25.1, cdc-25.2, cdc-25.3, cdc-25.4, cdk-1, his-1, his-2, his-3, his-4, his-5, his-6, his-7, his-8, his-11, his-16, his-17, his-18, his-24, his-25, his-31, his-32, his-35, his-37, his-38, his-39, his-40, his-41, his-42, his-45, his-47, his-48, his-55, his-57, his-58, his-59, his-60, his-61, his-62, his-63, his-64, his-65, his-66, his-67, his-68, his-69, his-70, his-71, his-72, his-73, his-74, rad-51. Other histone coding genes such as: his-9, -10, -12, -13, -14, -15, -19, -20, -21, -22, -23, -26, -27, -28, -29, -30, -33, -34, -36, -43, -44, -46, -49, -50, -51, -52, -53, -54, -56 showed no-expression in lab growth conditions. (TIF) [file pgen.1008338.s009.tif]

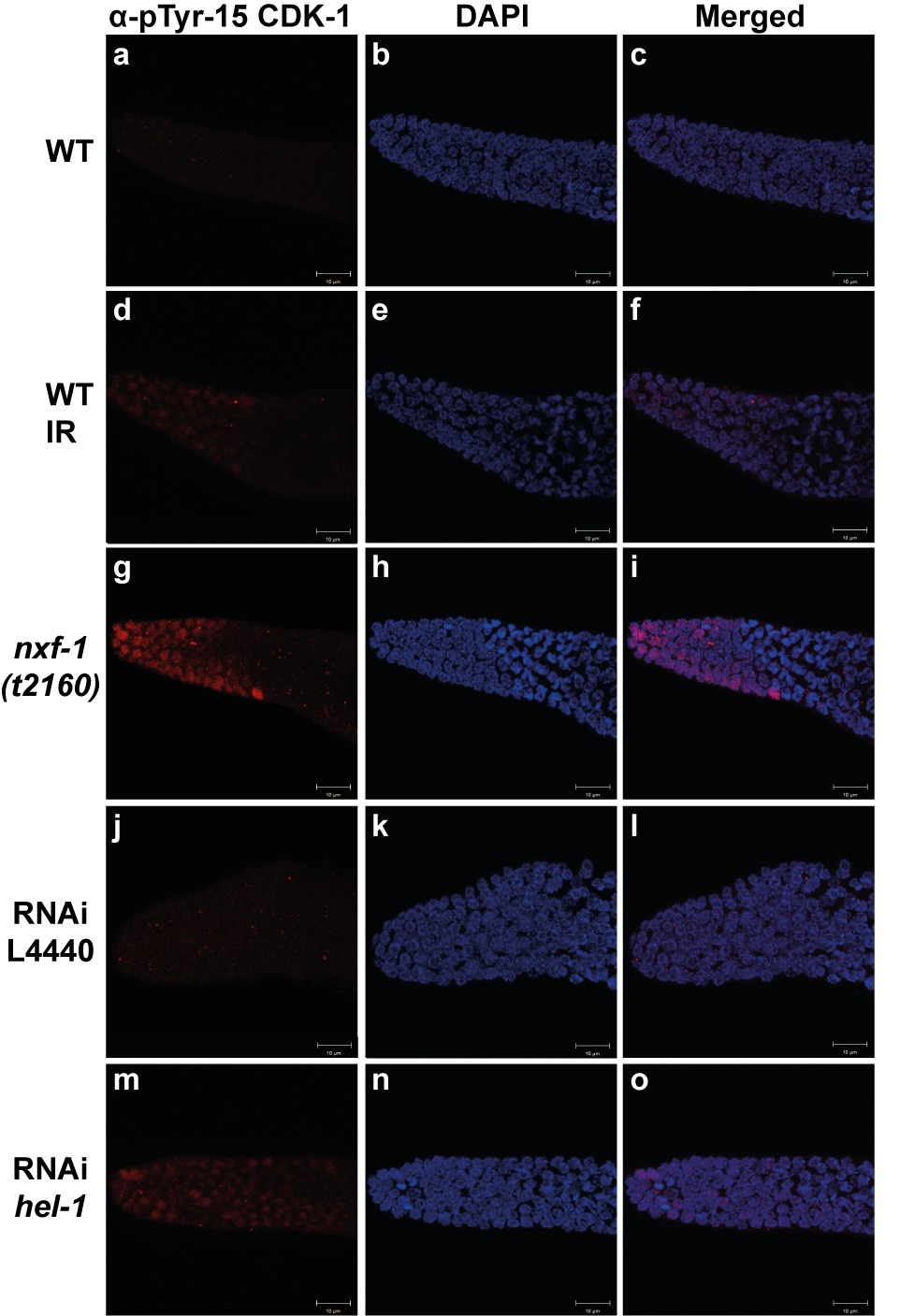

Supplement: S10 Fig — Mitotic cells in nxf-1(t2160ts) failed to proceed into mitosis and arrested at G2 phase (g, h and i), similar to gonads after IR (d, e and f) and depletion of hel-1 (m, n and o). N2 (WT) was used as the negative control (a, b and c). N2 (WT) and nxf-1(t2160ts) worms were synchronized. At the L4 stage, part of the N2 worms were fed the hel-1 bacterial RNAi and the bacterial RNAi clone of the empty L4440 vector was used as a control and fed to the rest of the worms (j, k and l). Another batch of L4 stage N2 worms were irradiated (90Gy). After 24 hours, gonads of nxf-1(t2160ts), N2 irradiated and non-irradiated worms, along with worms fed hel-1 and L4440, were dissected, fixed, immunostained with α-pTyr-15 CDK-1 and counterstained with DAPI. Scale bar: 10 μm. (TIF) [file pgen.1008338.s010.tif]

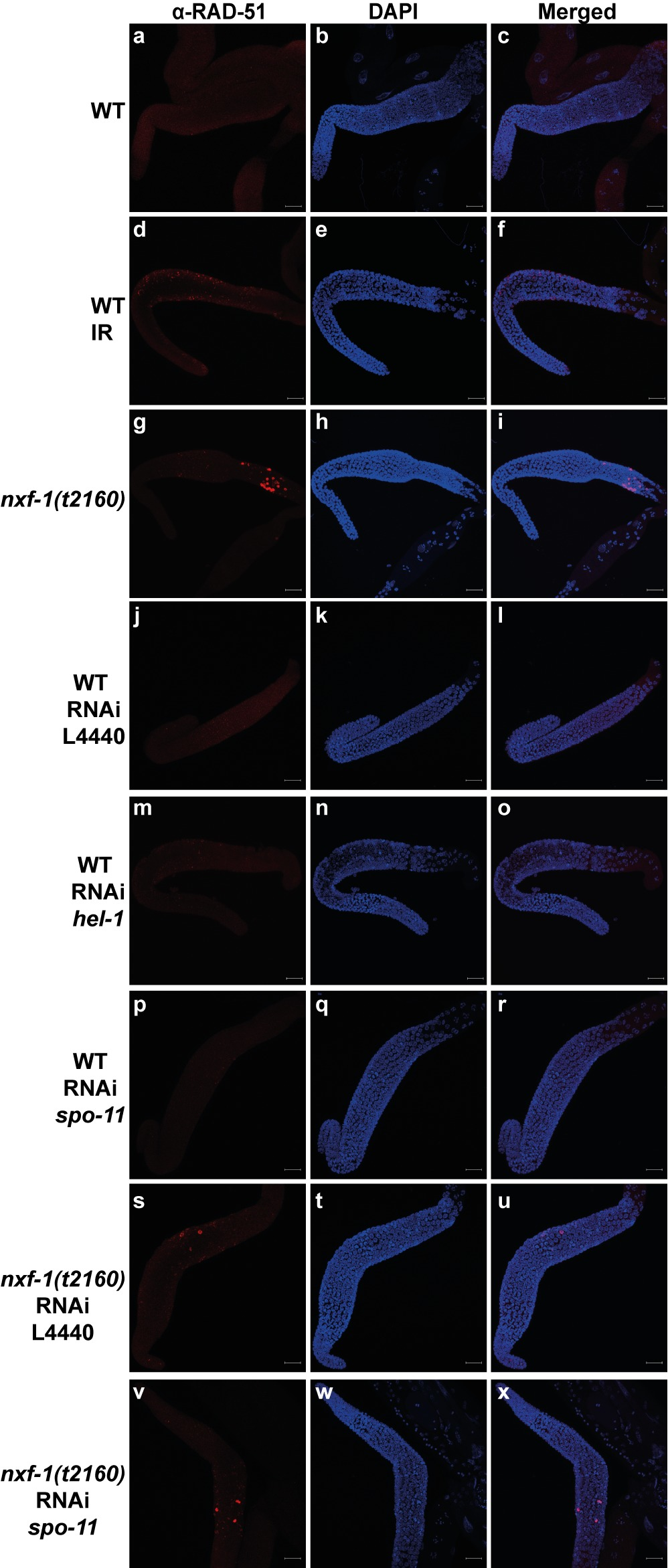

Supplement: S11 Fig — N2 (WT) and nxf-1(t2160ts) worms were synchronized. At the L4 stage, N2 (WT) worms were irradiated (90Gy). After 24 hours, gonads of N2 non-irradiated (a, b and c), N2 irradiated (d, e and f) and nxf-1(t2160ts) (g, h and i) worms were dissected, fixed, immunostained with α-RAD-51 and counterstained with DAPI. In another set of experiments, N2 (WT) and nxf-1(t2160ts) worms were synchronized and from the L1 stage, they were fed the spo-11 bacterial RNAi (p, q, r, v, w and x) and the empty L4440 vector (j, k, I, s, t and u) that was used as a control. At the L4 stage, a fraction of N2 (WT) worms were fed the hel-1 bacterial RNAi clones (m, n and o). One-day-old worms were dissected, and their gonads were fixed, immunostained with α-RAD-51 and counterstained with DAPI. Scale bar: 20μm. (TIF) [file pgen.1008338.s011.tif]

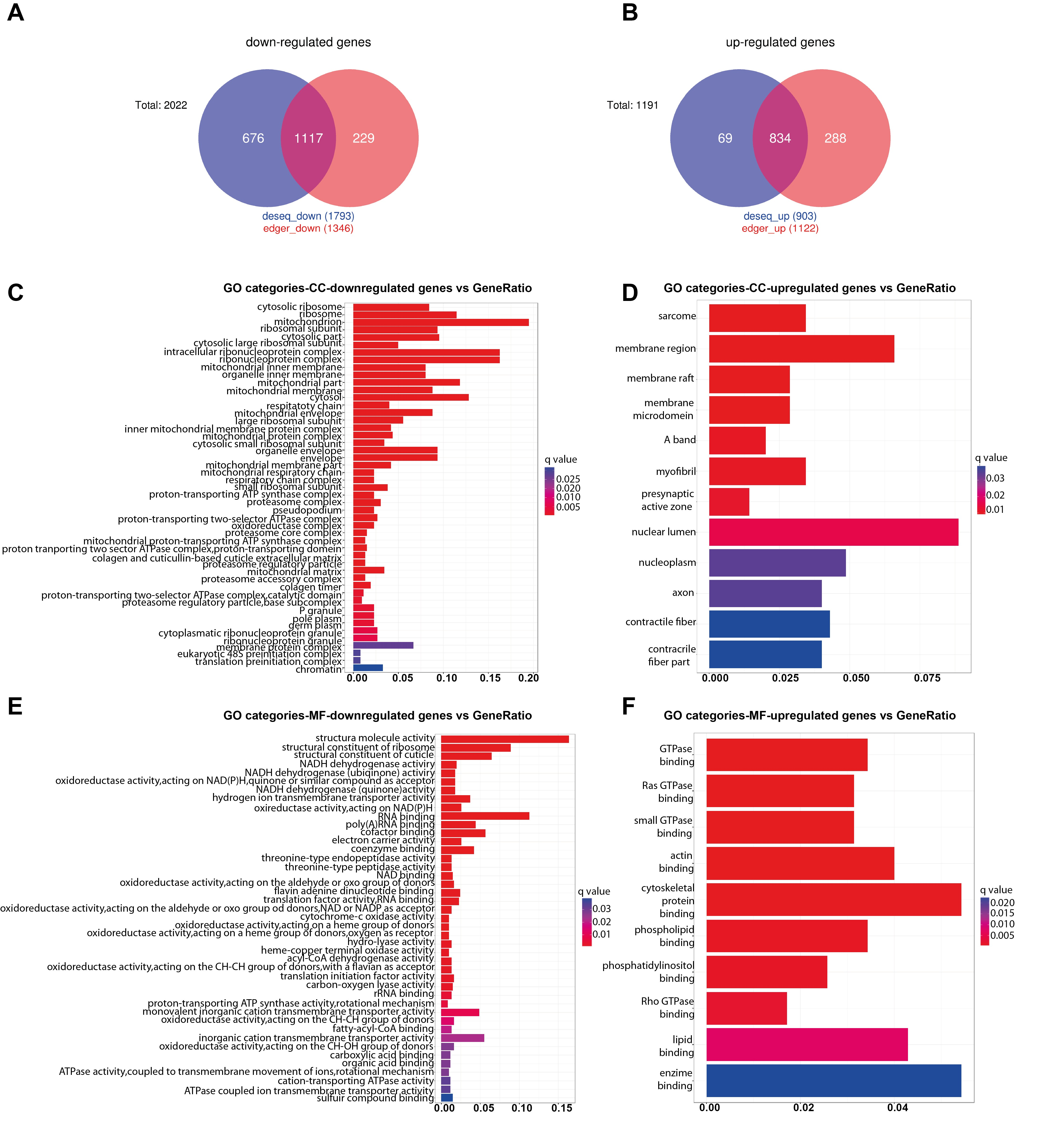

Supplement: S12 Fig — Statistical analysis with DeSeq and Edger shows 1117 downregulated (A) and 834 upregulated genes (B) in nxf-1(t2160ts) vs WT. Gene ontology (GO) analysis, cellular component (CC) analysis, and molecular function (MF) of differentially expressed downregulated and upregulated genes in nxf-1(t2160ts) vs. WT (C, D, E and F). The number of genes within each category is represented in color bars, one bar per GO term. Bar length indicates the number of genes belonging to the different GO categories and color indicates the statistical significance, from those with highly significant expression differences (red) to those with low expression differences (blue). (TIF) [file pgen.1008338.s012.tif]

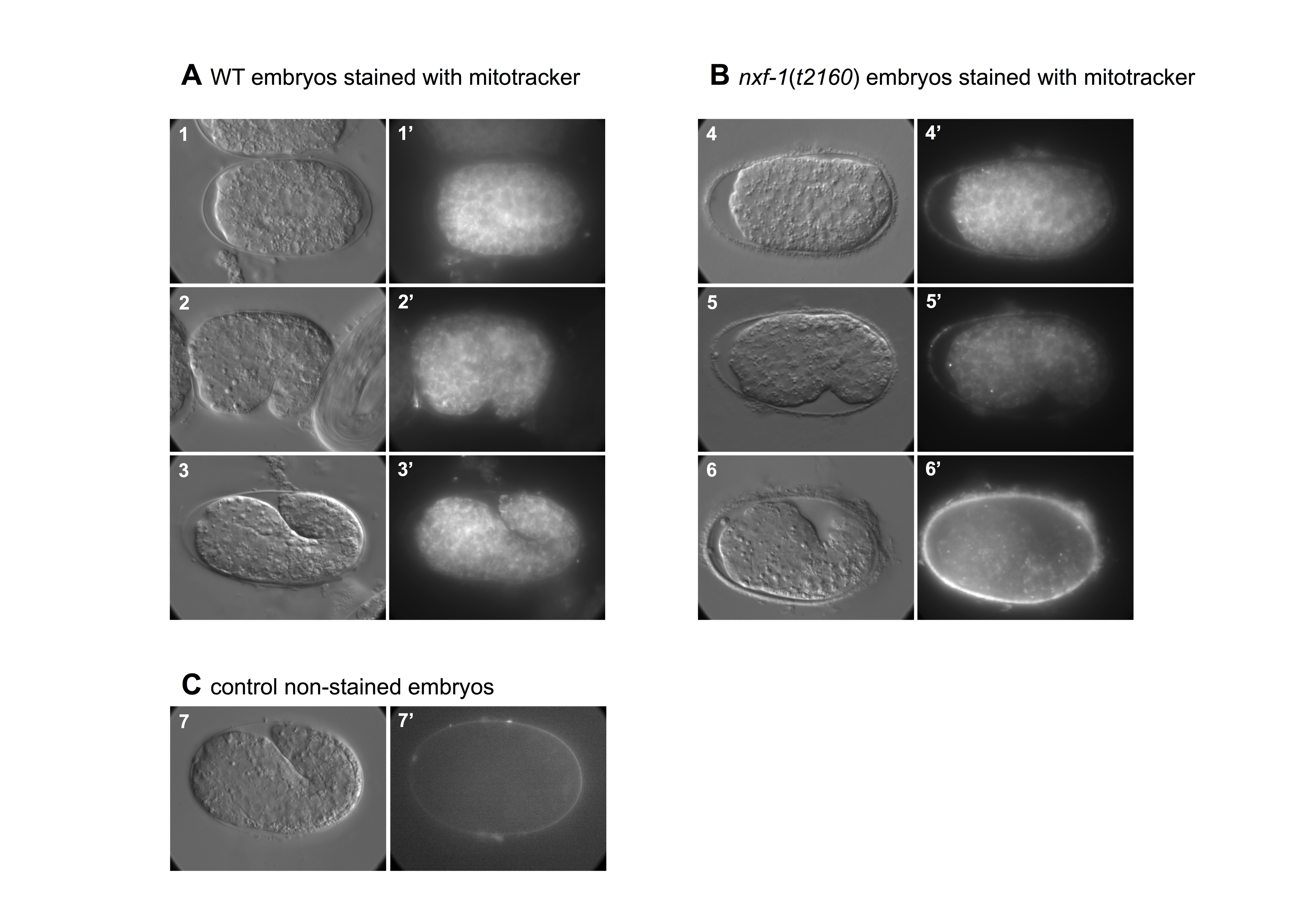

Supplement: S13 Fig — (A) WT embryos at different developmental stages (1–3) show a connected mitochondrial network in the cytoplasm of their cells visualized with Mitotracker staining (1'-3'). (B) nxf-1(t2160ts) mutant embryos at the same developmental stages (4–6) show a general dotted pattern of Mitotracker staining in the cytoplasm of their cells (4’-6’), indicating the additional presence of fragmented-type mitochondria. (C) shows a non-stained embryo as a control for autofluorescence (7–7’). (TIF) [file pgen.1008338.s013.tif]

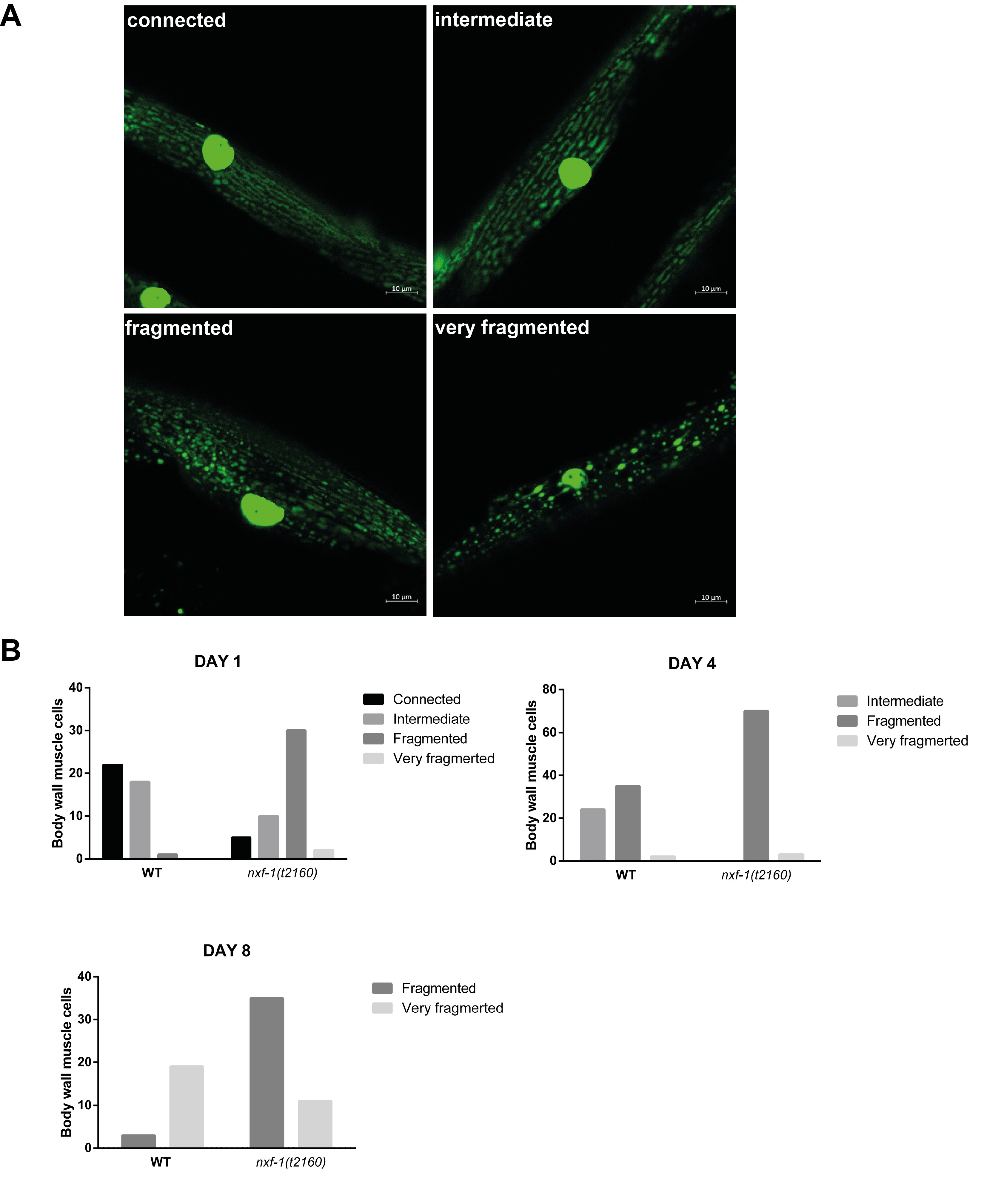

Supplement: S14 Fig — (A) Representative images of the different mitochondrial morphologies scored. (B) Transgenic animals expressing mitoGFP (ccIs4251 [(pSAK2) Pmyo-3::GFP::LacZ::NLS + (pSAK4) Pmyo-3::mitochondrial GFP + dpy-20(+)]) in body wall muscle cells (DAY 1, DAY 4 and DAY 8) were analyzed at different days after the L4 larval stage, respectively (DAY 1 WT n = 41 and nxf-1(t2160ts) n = 47; DAY 4 WT n = 61 and nxf-1(t2160ts) n = 73; DAY 8 WT n = 21 and nxf-1(t2160ts) n = 46). Because nxf-1(t2160ts) are ts, worms were grown at 15°C and then moved to 25°C at the L4 larval stage. Scale bar: 10μm. (TIF) [file pgen.1008338.s014.tif]

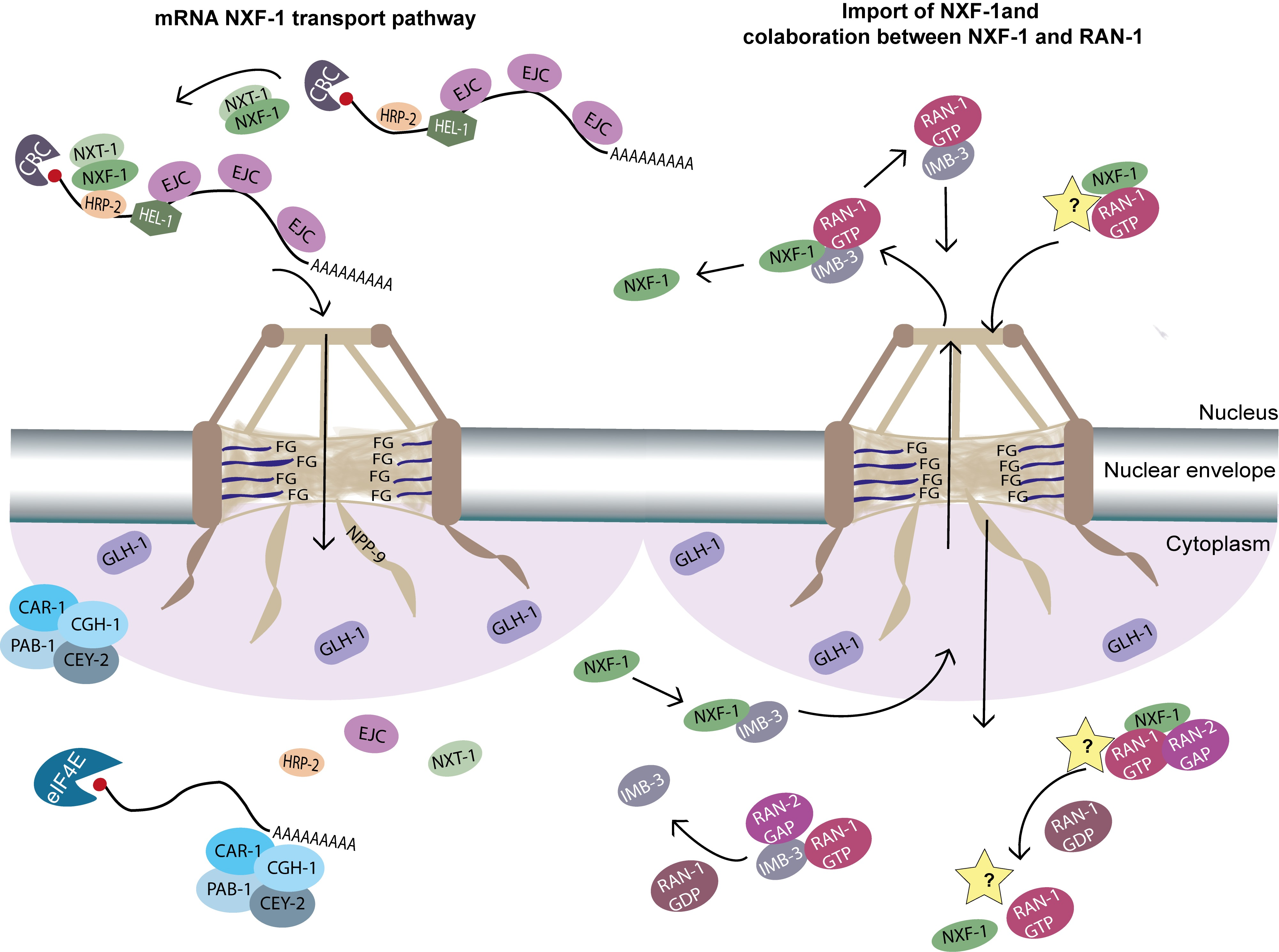

Supplement: S15 Fig — On the left-hand side, the main steps of mRNA export are shown: NXF-1/NXT-1 recruitment to the mRNP; mRNP export through the NPC where cytoplasmic fibril NPP-9 probably mediates the translocation step of mRNA across the NPC. GLH-1 probably mediates the release of mRNA from P granules into the cytoplasm; mRNAs are stored in P body particles. P body poly(A) binding protein PAB-1, decapping factor CGH-1, CAR-1/Rap55/Trailer hitch and Y-box protein CEY-2 are shown. Finally, import of NXF-1 from the cytoplasm to the nucleus via the IMB-3 transport in the RanGTPase-dependent pathway. On the right-hand side of the proposed model, NXF-1 exports unknown cargo using the RAN-1-dependent pathway. Once in the cytoplasm, RAN-2 associates with the cargo complex and this leads to its dissociation. (TIF) [file pgen.1008338.s015.tif]
