# Supplementary material for: Reduction of mRNA export unmasks different tissue sensitivities to low mRNA levels during Caenorhabditis elegans development
Source: PLoS Genet. 2019 Sep 16;15(9):e1008338. doi: 10.1371/journal.pgen.1008338 (PMC6762213; doi:10.1371/journal.pgen.1008338)
Supplement: S1 Table — (DOCX) [file pgen.1008338.s016.docx]

**Table S1. KEGG Pathway analysis.**

| **ID** | **Description** | **Pvalue** | **p.adjust** | **qvalue** | **Count** | **Condition** |
| --- | --- | --- | --- | --- | --- | --- |
| cel00010 | Glycolysis/Gluconeogenesis | 1.37E-06 | 3.00E-05 | 2.54E-05 | 18 | Downregulated |
| cel00020 | Citrate cycle (TCA Cycle) | 1.58E-06 | 3.00E-05 | 2.54E-05 | 16 | Downregulated |
| cel00071 | Fatty acid degradation | 0.00495 | 0.039594 | 0.033452235 | 15 | Downregulated |
| cel00190 | Oxidative phosphorylation | 3.14E-18 | 1.19E-16 | 1.01E-16 | 49 | Downregulated |
| cel00260 | Glycine, Serine and Threonine metabolism | 0.004919 | 0.039594 | 0.033452235 | 8 | Downregulated |
| cel00280 | Valine, Leucine and Isoleucine degradation | 0.00018 | 0.001952 | 0.001649068 | 16 | Downregulated |
| cel00562 | Inositol phosphate metabolism | 0.001477 | 0.021784 | 0.020209576 | 5 | Upregulated |
| cel00620 | Pyruvate metabolism | 2.64E-06 | 4.02E-05 | 3.40E-05 | 14 | Downregulated |
| cel00640 | Propanoate metabolism | 0.00521 | 0.039594 | 0.033452235 | 10 | Downregulated |
| cel03010 | Ribosome | 1.59E-26 | 1.21E-24 | 1.02E-24 | 53 | Downregulated |
| cel03013 | RNA transport | 0.000993 | 0.019535 | 0.01812306 | 13 | Upregulated |
| cel03015 | mRNA surveillance pathway | 0.00027 | 0.011875 | 0.011016861 | 10 | Upregulated |
| cel03050 | Proteasome | 2.31E-05 | 0.000293 | 0.000247482 | 16 | Downregulated |
| cel04020 | Calcium signaling pathway | 0.000403 | 0.011875 | 0.011016861 | 8 | Upregulated |
