# Supplementary material for: Reduction of mRNA export unmasks different tissue sensitivities to low mRNA levels during Caenorhabditis elegans development
Source: PLoS Genet. 2019 Sep 16;15(9):e1008338. doi: 10.1371/journal.pgen.1008338 (PMC6762213; doi:10.1371/journal.pgen.1008338)
Supplement: S2 Table — (DOCX) [file pgen.1008338.s017.docx]

**Table S2. Genes involved in the RNA transport and mRNA surveillance pathways, upregulated in *nxf-1(t2160)* compared to wild type.**

| **Gene ID** | **Gene Symbol** | **Description** | **KEGG pathway** |
| --- | --- | --- | --- |
| 190485 | Y65B4A.6 | ortholog of human EIF4A3 (eukaryotic translation initiation factor 4A3) | RNA transport/surveillance |
| 171696 | *smg-2* | Regulator of nonsense transcripts 1 | RNA transport/surveillance |
| 172418 | *smg-1* | nonsense-mediated mRNA decay | mRNA surveillance |
| 172864 | *rae-1* | mRNA transport factor | RNA transport |
| 179783 | *pnn-1* | PiNiN nuclear speckle-associated protein and splicing factor homolog | RNA transport/surveillance |
| 181473 | *pab-2* | Polyadenylated-binding protein, homolog with high similarity to human PABP 1 | RNA transport/surveillance |
| 171866 | *nxt-1* | NTF2-related export protein | RNA transport/surveillance |
| 191736 | *nxf-1* | Nuclear RNA export factor 1 | RNA transport/surveillance |
| 174232 | *npp-21* | Nuclear pore complex protein | RNA transport |
| 172813 | *npp-14* | Nuclear pore complex protein | RNA transport |
| 181044 | *eef-1A.2* | Elongation factor 1-alfa | RNA transport |
| 179760 | C44H9.4 | ortholog of human MOV10 (Mov10 RISC complex RNA helicase) | RNA transport/surveillance |
| 174546 | C05C10.2 | ortholog of human MOV10 (Mov10 RISC complex RNA helicase) | RNA transport/surveillance |
| 177735 | *aly-1* | Ref/ALY RNA export adaptor family | RNA transport/surveillance |
