# Supplementary material for: Reduction of mRNA export unmasks different tissue sensitivities to low mRNA levels during Caenorhabditis elegans development
Source: PLoS Genet. 2019 Sep 16;15(9):e1008338. doi: 10.1371/journal.pgen.1008338 (PMC6762213; doi:10.1371/journal.pgen.1008338)
Supplement: S3 Table — (DOCX) [file pgen.1008338.s018.docx]

**Table S4. *C. elegans* mutant and transgenic strains used in this study and some of their characteristics.**

| **Strain** | **Genotype** | **Characteristics** | **Source** |
| --- | --- | --- | --- |
| N2 | *C. elegans* wild isolate | Wild type | CGC |
| GE2440 | *unc-24 (e138)* IV*; dpy-11(e224); nxf-1(t2160)* V | Uncoordinated (unc), dumpy (dpy) and temperature sensitive. Maintained at 15°C. | Schnabel's lab |
| JCP495 | *nxf-1(t2160)*V | Temperature sensitive. Maintained at 15°C. | Our lab |
| CB4856 | Wild type. Low copy Tc1; pattern IX. *C. elegans* wild isolate. | Isolated from a pineapple field in Hawaii in 1972 by L. Hollen. Wild type. | CGC |
| RB1246 | *nxf-1(ok1281)* V/nT1 [qIs51] (IV;V) | C15H11.3 Heterozygotes are WT and GFP+. *ok1281* animals arrest as larvae. | CGC |
| QC47 | etIs1 [*ric-19*p::*ric-19*::GFP + *rol-6(su1006)* | Low levels of *ric19*p::GFP fusion protein expression driven by the ric-19 promoter in all neurons except for strong expression in the pharyngeal M2 neurons. | CGC |
| ML1735 | mcIs50 [*lin-26*p::*vab-10*(actin-binding domain)::GFP + *myo-2p*::GFP + pBluescript] | ABDVAB-10::GFP construct visualize actin with *myo-2*p::GFP marker. | CGC |
| YG1007 | baf-1(gk324) III/hT2 [bli-4(e937) let-?(q782) qIs48] (I;III); syls50 | syIs50 [cdh-3::GFP + dpy-20(+)]. Heterozygotes are WT and GFP+, and segregate arrested hT2 aneuploids, non-GFP gk324 homozygotes (Sterile and Unc). All worms expressed cdh-3::GFP at the anchor cell. qIs48 is an insertion of ccEx9747 with markers: myo-2::GFP expressed brightly in the pharynx throughout development, pes-10::GFP expressed in embryos, a gut promoter driving GFP in the intestine, and is homozygous lethal. | CGC |
| SM481 | pxIs10 [*pha-4*::GFP::CAAX + (pRF4) *rol-6(su1006)*] | Roller line that has GFP localized to the plasma membrane of the pharynx, gut and rectal cells in embryos and the somatic gonad during the L2-L3 larval stage and beyond. | CGC |
| OP37 | *unc-119(ed3)* III; wgIs37 [*pha-4*::TY1::EGFP::3xFLAG + *unc-119*(+)] | TY1::EGFP::3xFLAG tag inserted in-frame at the C-terminus of the *pha-4* coding sequence. | CGC |
| FT828 | *unc-119(ed3)* III; xnIs312 [*par-6*p::*par-6*::mCherry + *unc-119*(+)] | Expresses *par-6*::mCherry maternally and zygotically. Expression is present in many cells, including early embryos, epithelial cells, the excretory cell, and the germ line. | CGC |
| ST65 | ncIs13[ajm-1::GFP] | AJM::GFP expressed in CeAJs. | CGC |
| SU295 | jcIs25 [pPE103 (*jac-1*::GFP) + *rol-6*(*su1006*)] | Rollers. | CGC |
| FT250 | xnIs96 [pJN455(*hmr-1*p::*hmr-1*::GFP::*unc-54* 3'UTR) + *unc-119*(+)] | GFP starts in membranes at the ~100 cell stage, becomes apically bright in older embryos and larvae; most prominent in nerve ring in adults. | CGC |
| TH502 | ddIs290 [*sax-7*::TY1::EGFP::3xFLAG(92C12) + *unc-119*(+)] | TY1::EGFP::3xFLAG tag inserted in-frame at the C-terminus of the coding sequence by recombineering. Expression of transgene confirmed by GFP. | CGC |
| SU265 | jcIs17 [*hmp-1*p::*hmp-1*::GFP + *dlg-1p*::*dlg-1*::dsRed + *rol-6*(*su1006*)] | Roller. | CGC |
| SD1347 | ccIs4251 [(pSAK2) *myo-3*p::GFP::LacZ::NLS + (pSAK4) *myo-3*p::mitochondrial GFP + dpy-20(+)] I | myo-3p::mitochondrial GFP. | CGC |
| JCP484 | *nxf-1(t2160)* V; pxIs10 [*pha-4*::GFP::CAAX + (pRF4) *rol-6(su1006)*] | Contains *nxf-1(t2160)* V; pxIs10. Temperature sensitive. Grown at 15°C to maintain. | Our lab |
| JCP561 | *unc-119(ed3)* III; *nxf-1(t2160)* V; wgIs37 [*pha-4*::TY1::EGFP::3xFLAG + *unc-119*(+)] | Contains *nxf-1(t2160)* V; wgIs37. Temperature sensitive. Maintained at 15°C. | Our lab |
| JCP567 | *nxf-1(t2160) V;* etIs1 [*ric-19*p::*ric-19*::GFP + *rol-6(su1006)*] | Contains *nxf-1(t2160)* V; etIs1. Temperature sensitive. Maintained at 15°C. | Our lab |
| JCP510 | *nxf-1(t2160) V; syls50[cdh-3::GFP + dpy-20(+)]* | Contains *nxf-1(t2160)* V; etIs1. Temperature sensitive. Maintained at 15°C. | Our lab |
| JCP543 | *nxf-1(t2160)* V; ncIs13[ajm-1::GFP] | Contains *nxf-1(t2160)* V; ncIs13. Temperature sensitive. Maintained at 15°C. | Our lab |
| JCP511 | *nxf-1(t2160)* V; mcIs50 [*lin-26*p::*vab-10*(actin-binding domain)::GFP + *myo-2p*::GFP + pBluescript] | Contains *nxf-1(t2160)* V; mcIs50. Temperature sensitive. Maintained at 15°C. | Our lab |
| JCP489 | *nxf-1(t2160)* V; jcIs25 [pPE103 (*jac-1*::GFP) + rol-6(su1006)] | Contains *nxf-1(t2160)* V; jcIs25. Temperature sensitive. Maintained at 15°C. | Our lab |
| JCP486 | *nxf-1(t2160)* V; xnIs96 [pJN455(*hmr-1*p::*hmr-1*::GFP::*unc-54* 3'UTR) + *unc-119*(+)] | Contains *nxf-1(t2160)* V; xnIs96. Temperature sensitive. Maintained at 15°C. | Our lab |
| JCP506 | *nxf-1(t2160)* V; ddIs290 [*sax-7*::TY1::EGFP::3xFLAG(92C12) + *unc-119*(+)] | Contains *nxf-1(t2160)* V; ddIs290. Temperature sensitive. Maintained at 15°C. | Our lab |
| JCP485 | *nxf-1(t2160)* V; jcIs17 [*hmp-1*p::*hmp-1*::GFP + *dlg-1p*::*dlg-1*::dsRed + *rol-6*(su1006)] | Contains *nxf-1(t2160)* V; jcIs17. Temperature sensitive. Maintained at 15°C. | Our lab |
| JCP565 | *nxf-1(t2160)* V; ccIs4251 [(pSAK2) *myo-3*p::GFP::LacZ::NLS + (pSAK4) *myo-3*p::mitochondrial GFP + dpy-20(+)] | Contains *nxf-1(t2160)* V; ccIs4251. Temperature sensitive. Maintained at 15°C. | Our lab |
| JCP519 | *nxf-1(t2160)* V; jcpEx6[pAZ09(*nxf-1p*::*nxf-1*::3xFLAG::eGFP::*nxf-1*UTR] | Contains *nxf-1(t2160)* V; jcpEx6. Maintained at 25°C. | Our lab |
| JCP633 | *unc-119(ed3)* III; *nxf-1(t2160)* V; xnIs312 [*par-6*p::*par-6*::mCherry + *unc-119*(+)] | Contains *nxf-1(t2160)* V; xnIs312. Maintained at 15°C. | Our lab |
